# Comprehensive Characterization of Metabolism-Associated Subtypes of Renal Cell Carcinoma to Aid Clinical Therapy

**DOI:** 10.1155/2022/9039732

**Published:** 2022-02-27

**Authors:** Zhixian Yao, Zhong Zheng, Xinyi Zheng, Hantao Wu, Weiguang Zhao, Xingyu Mu, Feng Sun, Ke Wu, Junhua Zheng

**Affiliations:** ^1^Department of Urology, Shanghai General Hospital, Shanghai Jiao Tong University, School of Medicine, Shanghai, China; ^2^Department of Urology, Renji Hospital, Shanghai Jiao Tong University School of Medicine, Shanghai, China; ^3^Department of Pharmacy, Huashan Hospital, Fudan University, Shanghai, China

## Abstract

Renal cell carcinoma (RCC) is a disease characterized by excessive administration complexity because it exhibits extraordinary nonuniformity among distinct molecular subtypes. We herein intended to delineate the metabolic aspects of clear cell RCC (ccRCC) in terms of the gene expression profile. Recent studies have revealed that metabolic variations within tumors are related to the responsiveness to immune checkpoint inhibitor (ICI) therapy and patient prognosis. We used 100 previously reported metabolic (MTB) pathways to quantify the metabolic landscape of the 729 ccRCC patients. Three MTB subtypes were established, and the MTB scores were calculated using principal component analysis (PCA). The high MTB score group had better overall survival (OS) and was associated with higher expression of immune-checkpoint and immune-activity signatures. The opposite was true of the low MTB score group, which may explain the poor prognosis of these patients. Three ICI-treated cohorts or tyrosine kinase inhibitor (TKI) treated cohort proved that patients with higher MTB scores exhibited notable therapeutic benefits and clinical gains. This research explained that the MTB score could be applied as a powerful prognostic indicator and predictive of ICI or TKI therapy. Assessing the MTB scores in a more extended group will facilitate our perception of tumor metabolism and provide guidance for studies on targeted approaches for ccRCC patients.

## 1. Introduction

Renal cell carcinoma (RCC) remains in the top 10 most commonly diagnosed malignancies globally [1]. Accounting for 70% of pathologically determined RCC, clear cell renal cell carcinoma (ccRCC) is often histologically marked by enriched lipid and glycogen infiltration [2]. Due to the insidious and asymptomatic onset of the disease, approximately 30% of patients have already developed metastatic disease when diagnosed, and another 20% of patients with early-stage RCC progress to metastatic RCC (mRCC) despite of initial treatment which eventually results in a 5-year survival rate of only 12% [3–5].

RCC is one of the most investigated and perhaps the representative of human cancers distinguished by metabolic reprogramming which is evident in various systemic manifestations [6, 7]. A number of findings have unveiled various metabolic changes that are directly or indirectly involved throughout cancer development. Despite the widely accepted Warburg effect and glutamine addiction, upregulation of glutamine metabolism and lipid synthesis, and reductive carboxylation actively arises in many RCC cells, which enables tumor cells to swiftly reproduce [8–11]. Therefore, researchers have worked on strategies to classify ccRCC patients into different risk groups by tumor metabolic patterns. On account of transcriptome and metabolic analysis of ccRCC, Zhao et al. have concluded a gene signature based on fatty acid metabolic enzymes that could divide ccRCC patients into distinct subgroups [12]. Another study integrated transcriptomic and metabolomic analyses revealed that disordered metabolism of succinate, beta-alanine, purines, glucose, and myo-inositol may contribute to the unfavorable prognosis of RCC patients [13].

Cancer cells rewire their metabolism, which influences the representation of specific cell markers and intervenes in the immune response in the tumor microenvironment (TME) [14, 15]. Ramapriyan et al. have concluded that the PI3K-Akt-mTOR axis, hypoxia-inducible factor pathway (HIF), adenosine pathway, Janus kinase/signal transducers and activators of transcription (JAK/STAT) pathway, Wnt/Beta-catenin signaling, and amino acid depletion metabolic pathways are essential metabolic signaling modulating TME and resulting immune checkpoint inhibitor (ICI) resistance in various malignancies [16].

Several studies have shown that various metabolic features within cancers may affect therapeutic strategies. In a breast cancer research, early-stage patients were treated with N-acetylcysteine to subdue oxidative stress and reduce tumor growth. And the Monocarboxylate transporter 4 (MCT4) which serves as a lactate exporter was found to be decreased in stromal cells after N-acetylcysteine treatment, together with decreasing proliferative contents [17]. While in melanoma, Renner et al. discovered that patients with higher level of glycolysis displayed an inferior response to ICI therapy [18]. Similar findings have also been reported in RCC where higher glucose intake within tumors is associated with low T cell infiltration and poor response to ICI treatment [19, 20].

Recent advances in ICI cancer treatment have shed light on RCC patients, but the response rate remains low, and most patients are resistant to ICI therapy [21, 22]. Though numerous strides have been performed to identify the metabolic mechanism and optimal treatment of ccRCC progression, molecular subtypes affecting the ccRCC patient survival as well as ICI treatment response are still inadequately understood [23]. In this research, we investigated three ccRCC cohorts that were combined to form a dataset of 729 cases for consensus clustering reflecting on metabolic pathways. Two further processed microarray data sets with 348 and 56 samples were used to evaluate the ICI treatment response and 53 samples for targeted therapy in the two MTB subtypes, respectively. We aimed to explore the metabolic activities among individual ccRCC patients, which can help identify patients with distinguished clinical outcomes and guide personalized medical treatment.

## 2. Materials and Methods

### 2.1. Acquiring RCC Datasets and Samples

Public data repositories, namely, the Cancer Genome Atlas (TCGA, http://cancergehttp://nome.nih.gov/), Gene Expression Omnibus (GEO, http://www.ncbi.nlm.nih.gov/geo/), and the Array Express Database (AED, https://www.ebi.ac.uk/arrayexpress/), were explored for usable datasets for ccRCC. Cohorts without enough sample size (<100) or relevant clinical records without overall survival (OS) data were filtered out. The RNA sequencing data (value: raw counts) of TCGA-KIRC and CTPAC-3 datasets were downloaded from the Genomic Data Commons Data Portal (https://portal.gdc.cancer.gov/). The ccRCC microarray dataset E-MTAB-1980 from the University of Tokyo, E-MTAB-3218 from Bristol-Myers Squibb clinical trial, and E-MTAB-3267 from Paris Descartes University were downloaded from the AED [24–26]. For all microarray datasets (E-MTAB-1980, E-MTAB-3267, and E-MTAB-3218), raw data (.CEL Intensity files) were downloaded, and Robust Multi-array Average (RMA) [27] algorithm was used to adjust the background. This step was conducted by the *rma* function from the Affy R package which sequentially performed background processing, log2 transformation, quantile normalization, and probe expression calculation. For the same gene matched by multiple probes, the expression level was calculated by the median value of all probes. To form the integrated dataset, the RNA-Seq data of TCGA-KIRC and CTPAC-3 datasets were converted into transcripts per kilobase million (TPMs), which were more comparable to those measured from microarrays [28]. Further, the “ComBat” function from the SVA R package was operated to rectify the intrinsic batch effects among multicohorts [29].

### 2.2. Consensus Clustering for Metabolic Pathways

A total of 100 metabolism pathway signatures were obtained from earlier issued studies [30, 31]. In addition, we applied the single-sample GSEA (ssGSEA) method for the evaluation of enrichment degree of the metabolism-related signatures via GSVA R package [32]. For the consensus clustering analysis, the *K*-means method was conducted to determine the optimal MTB clusters using the “ConsensuClusterPlus” R package [33]. This procedure was based on Euclidean distance and Ward's linkage which was resampled for 1000 times to ensure the robustness. We used Microenvironment Cell Populations-counter (MCP-counter) algorithm to calculate infiltration levels for eight immune and 2 stromal cell populations in each sample [34]. The enrichment of stromal and immune scores of each individual was calculated using the ESTIMATE method [35]. Previously reported four ICI-resistance-related gene sets were extracted from the Molecular Signatures Database (MSigDb) and quantified via GSVA R package [16, 36].

### 2.3. DEGs Correlated to the Corresponding MTB Subtypes

Individuals were sorted into the MTB clusters in terms of their distinguished metabolic enrichment scores. The LIMMA R package was employed to identify the genes correlated to the MTB clusters. Differentially expressed genes (DEGs) among different MTB subtypes were thought to be significant when the adjust *p* value was < 0.05, and the corresponding fold-change was > 1.3.

### 2.4. Reduced Dimension and Construction of MTB Score

Primarily, hierarchy clustering was applied to classify the patients in TCGA-KIRC cohort with their inherited DEG values. Besides, DEGs that show the positive correlation with the MTB clusters were defined as the MTB gene signature A and negative correlation with the MTB gene signature B. Subsequently, the Boruta algorithm was operated to further reduce the dimension of the MTB gene signatures which were interpreted by the R package clusterProfiler [37, 38]. We also used clusterProfiler to draw an enrichment map for the corresponding gene set which is a network-based process for gene-set enrichment visualization and explanation [39]. The first principal component was derived to generate the signature score by using the principal component analysis (PCA) algorithm. Finally, we implemented a process identical to the gene expression grade index [40] to define the MTB score of each subject:

(1) $$\begin{array}{c} \text{MTB Score}=\sum \text{P}\text{C}1_{\text{B}}-\sum \text{P}\text{C}1_{\text{A}}. \end{array}$$

### 2.5. Acquisition of Genomic Mutation Data

The homologous gene somatic mutation data (MAF files) of subjects in the TCGA-KIRC project were obtained from the GDC data portal. To calculate the tumor mutation burden (TMB) of each ccRCC, we considered all nonsynonymous mutations into account. The top 20 cancer-driving somatic variants were estimated in high and low MTB groups by employing the “maftool” R package [41], respectively.

### 2.6. Comparing the Response of Different MTB Groups from ICI or TKI Therapy

To assess the clinical value of the MTB scores, the IMvigor210 cohort with 348 metastatic urothelial carcinoma cases treated with atezolizumab was downloaded freely from http://research-pub.gene.com/IMvigor210CoreBiologies [42]. And another two datasets, E-MTAB-3267 with 53 metastatic ccRCC cases treated by sunitinib and E-MTAB-3218 with 56 metastatic ccRCC cases treated with nivolumab, were downloaded from the AED. The above three independent cohorts that received ICI or TKI therapy were investigated to validate the MTB scores. Further, we estimated the TKI agent response for each individual based on the Genomics of Drug Sensitivity in Cancer (GDSC). Three regularly used TKI drugs in RCC treatment—sorafenib, pazopanib, and axitinib—were picked. And the samples' half-maximal inhibitory concentration (IC50) of the samples was calculated via ridge regression and was 10-fold cross-validated using the R package “pRRophetic.”

### 2.7. Statistical Analysis

All the calculations and statistical analyses were conducted in the R language environment (R version 4.0.3). The Kruskal-Wallis examination was employed for comparisons larger than two groups, and the Wilcoxon *t*-test was applied for comparisons with two collections. Categorical variables were analyzed using the Chi-square test or Fisher's exact test when the theoretical frequency was <5. To divide patients into high and low MTB scores, respectively, the survival R package was applied to reach the maximum select rank statistic (MSRS). Survival analysis was conducted by Kaplan–Meier procedures, and statistical significance was examined by the log-rank test. Correlation analysis was performed using the Spearman method. Statistical significance was defined as a two-tailed *p* value < 0.05.

## 3. Results

### 3.1. Identifying Three MTB Subtypes and Their Distinct Immune Infiltration Patterns

**Figure 1:**
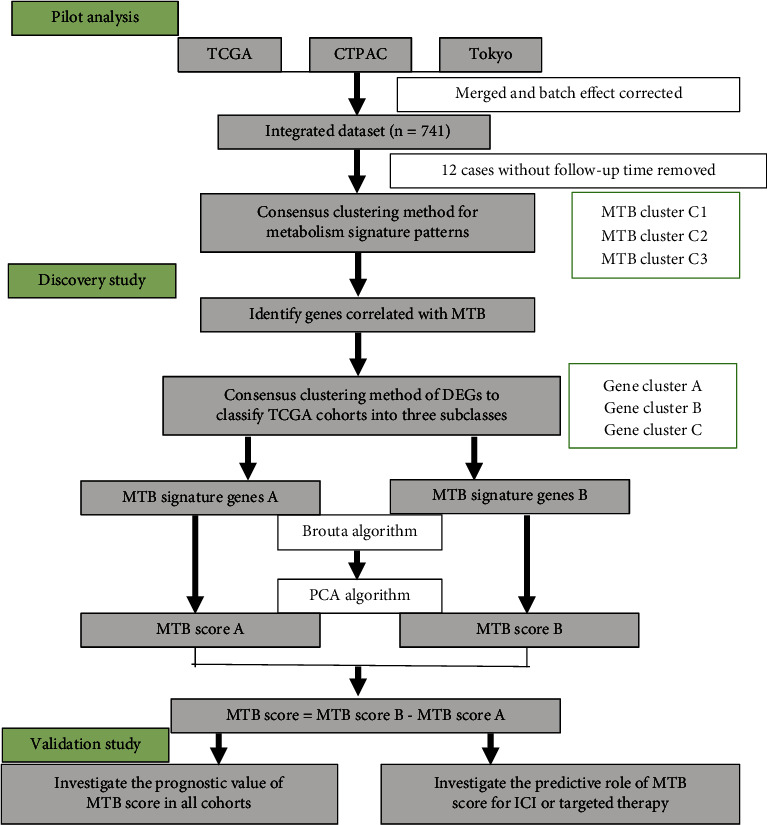
Flowchart of the study design.

The overall design of the study is presented in the flowchart (Figure 1), and the baseline characteristics of all ccRCC cases from different groups are displayed in Table 1. To form the integrated dataset (TCGA; CTPAC-3; Tokyo), the SVA R package was applied to rectify the batch effect (Figure S1A and S1B). ssGSEA scores of the previously recorded 100 metabolic pathways were adopted as the basis for consensus clustering analysis (Figure S1C and S1D).

**Figure 2:**
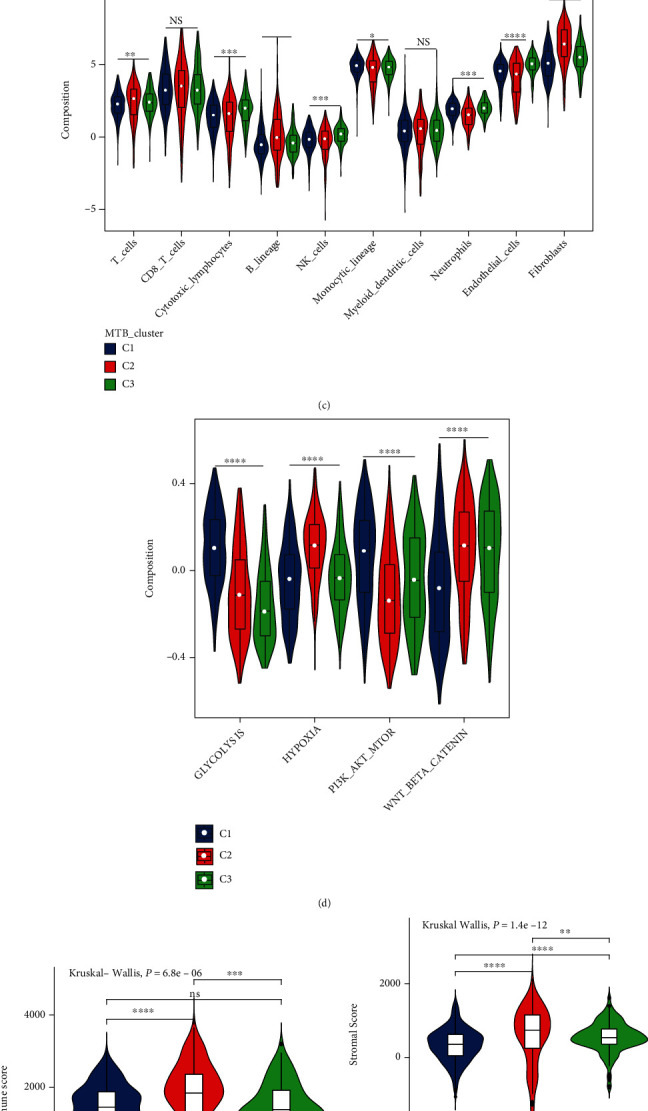
Classification of ccRCC clusters on the metabolism-associated pathways and immune infiltration patterns among clusters. (a) Consensus clustering of 100 metabolic signalings in three independent ccRCC groups. Rows serve as metabolic pathways, and columns serve as individuals. (b) Kaplan-Meier analysis for overall survival (OS) of the integrated dataset in three MTB clusters. The Log-rank *p* value was showed < 0.001, HR_(C2 − C3)_ = 2.3, HR_(C1 − C3)_ = 1.1. ((c) and (d)) The portion of immune infiltration fractions and four ICI resistance associated pathways in three MTB clusters. We also plotted the immune score and stromal score of three MTB clusters. ((e) and (f)) The difference in the stromal score and immune score among distinct MTB clusters. The Kruskal-Wallis test was applied to determine the statistical difference in three MTB clusters. (g) GSEA analysis unveils discrete enriched upregulated and downregulated gene sets among three subtypes. Rows are determined by the elected 30 gene sets and columns by consensus summaries for all subtype. All gene sets are marked by distinct colors. (^∗^*p* < 0.05; ^∗∗^*p* < 0.01; ^∗∗∗^*p* < 0.001; ^∗∗∗∗^*p* < 0.0001).

In total, 729 samples of kidney cancer were initially available for analysis, and three distinct MTB subtypes with varied overall survival (OS) patterns were identified (log-rank *p* value < 0.001; Figures 2(a) and 2(b)). Within three MTB clusters, the C1 cluster is marked by higher level of norepinephrine biosynthesis and vitamin B6 metabolism and possesses a better prognosis with a median follow-up time of 50.7 months (median survival time (MST) not reached). The C2 cluster is symbolized by a lower level of biosynthesis of adrenaline and amino acid metabolism and harbors an awful OS (MST: 57.1 months). No specific metabolic pathways were identified in the C3 cluster according to the differential analysis, and the OS was relatively better (MST: 118.5 months). Given that cells in the TME closely interact with tumor metabolism, we compared the immune and stromal cell composition in TME among different MTB clusters. We found the C1 cluster demonstrated a significantly higher density of monocytic lineage cells and neutrophils and while the C3 cluster showed higher levels of endothelial cells and natural killer (NK) cells (Figure 2(c)). A higher density of NK cells in tumor samples reflected enhanced cytotoxic capabilities in RCC which may give clues to its better prognosis [43]. And the C2 cluster was associated with higher counts of total T cells, B lineage cells, and fibroblasts, as well as higher immune and stromal scores, respectively (Figures 2(c), 2(e), and 2(f)). Similar to previous research, elevated infiltration level of fibroblasts and stromal scores was proved to suppress antitumor immunity and weaken the immunotherapy response in various cancers [44–46]. Furthermore, differences in ICI-resistance associated pathways were also observed, with C1 displaying a significantly higher score on glycolysis and PI3K-Akt-mTOR axis and C2 showing elevated composition on hypoxia and Wnt/Beta-catenin signaling (Figure 2(d)). To identify gene sets enriched within individual subtypes, we next conducted GSEA analysis. We then selected the top 10 most significant gene sets for each subtype to build a pathway heat map, which explained discrete gene sets enriched in each subclass (Figure 2(g)).

### 3.2. Discovering Metabolic Gene Subtype

**Figure 3:**
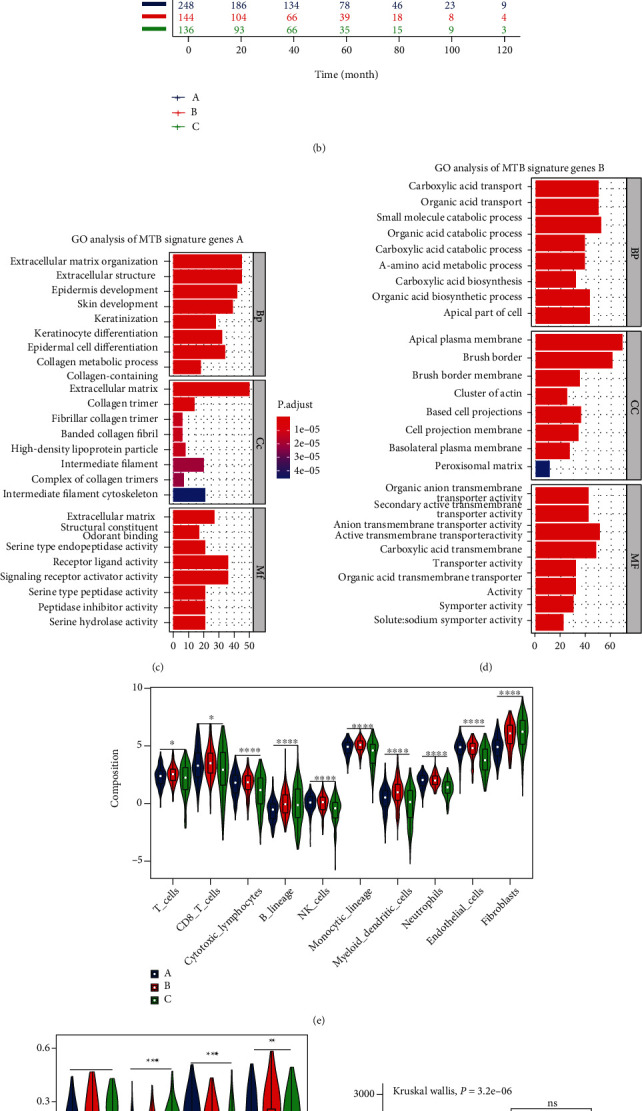
Classification of metabolic gene subclasses. (a) Consensus clustering of shared DEGs among three MTB cluster groups to classify cases into three combinations: gene clusters A–C. (b) Kaplan-Meier analysis for overall survival (OS) of the integrated dataset in three MTB gene clusters. The Log-rank *p* value was < 0.001, HR_(B − A)_ = 2.3, HR_(C − A)_ = 1.2. ((c) and (d)) Gene Ontology (GO) enrichment analysis of the two MTB-relevant signature genes: MTB signature genes A and B. The *x* axis represents the number of genes within per GO term. BP: biological process; CC: cellular component; MF: molecular function. ((e) and (f)) The portion of immune infiltration fractions and four ICI resistance-associated pathways in three MTB gene clusters. We also plotted the immune score and stromal score of three MTB gene clusters. ((g) and (h)) The difference in the stromal score and immune score among distinct MTB gene clusters. The Kruskal-Wallis test was applied to determine the statistical difference in three MTB gene clusters. (^∗^*p* < 0.05; ^∗∗^*p* < 0.01; ^∗∗∗^*p* < 0.001; ^∗∗∗∗^*p* < 0.0001).

To interpret the potential biological features of distinct metabolic subtypes, the LIMMA package in R was applied for differential analyses to discover the transcriptome alterations among these subclasses. In the ensuing investigation, we focused mainly on the TCGA RCC cohort, which possessed the most comprehensive genomic and clinical message. We conducted the consensus clustering of 1,201 differentially expressed genes (DEGs) (Figure S2D), collected from differential analysis among three MTB clusters, which then organized the TCGA group into three cohorts: gene clusters A–C (Figure S2A, S2B and S2C). Therefore, we defined 533 gene signatures positively related to the gene cluster as the MTB gene signature A, and the remainder of the DEGs were defined as the MTB gene signature B (Table S1). Subsequently, to further reduce the redundancy of genes, we adopted the Boruta algorithm for feature selection in the MTB gene signatures A and B, and the heat map described 206 most representative genes at the end (Figure 3(a) and Table S2). Enriched gene ontology terms including biological process (BP), cellular component (CC), and molecular function (MF) are presented in Figures 3(c) and 3(d).

For survival analysis among the three gene clusters, cases in gene cluster A demonstrated favorable outcomes with a median follow-up time of 50.8 months (MST not reached) and exhibited higher levels of neutrophils, endothelial cells, and PI3K-Akt-mTOR axis and lower stromal and immune scores than those in cluster C (Figures 3(b) and 3(e)–3(h)). And cases in gene cluster B showed a median OS (MST: 118.5 months) and were associated with higher counts of total T cells, CD8^+^ T cells, cytotoxic lymphocytes, B lineage, NK cells, monocytic lineage, and myeloid dendritic cells (Figures 3(b) and 3(e)). As for cluster C, which harbors the poorest OS (MST: 57.1 months) exhibited an increased stromal fibroblasts infiltration and higher scores in ICI-resistance associated pathways including hypoxia and Wnt/Beta-catenin signalings (Figures 3(b) and 3(e)–3(h)). Interestingly, we found gene cluster A showed an increased score on the PI3K-Akt-mTOR axis though possessing a comparatively better OS (Figure 3(f)).

### 3.3. Pathway Interactions and Installation of the MTB Score

**Figure 4:**
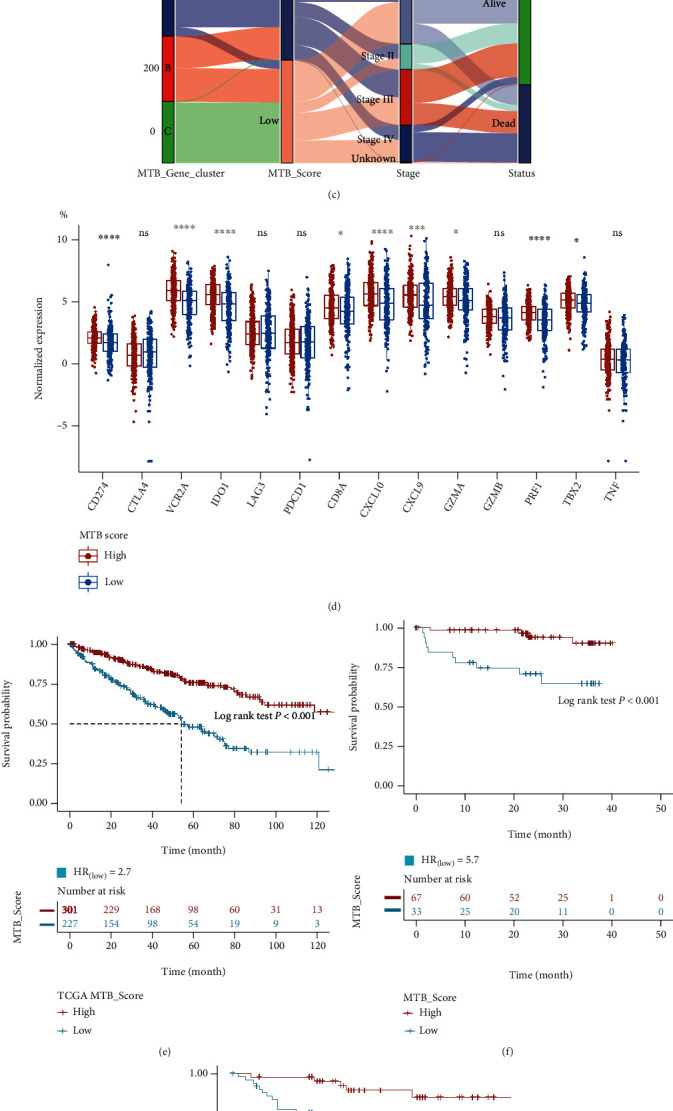
Pathway interactions and construction of the MTB score. (a) Enrichment map analysis of KEGG pathways among three gene clusters. The size of the circle represents the count of genes. (b) Comparison of enriched KEGG pathways between two MTB gene signatures. The size of the circle represents the count of genes. (c) Alluvial chart of MTB gene cluster division within groups with different MTB clusters, MTB scores, and survival endings. (d) Variation of immune checkpoint genes (CD274, CTLA4, HAVCR2, IDO1, LAG3, and PDCD1) and immune function genes (CD8A, CXCL10, CXCL9, GZMA, GZMB, PRF1, TBX2, and TNF) in high and low MTB score subgroups. (e) Survival analysis for patients with high and low MTB scores in the TCGA RCC cohort. HR_(low MTB score)_ = 2.7, Log-rank *p* value < 0.001. (f) Survival analysis for patients with high and low MTB scores in the CTPAC-3 RCC cohort. HR_(low MTB score)_ = 5.7, Log-rank *p* value < 0.001. (g) Survival analysis for patients with high and low MTB scores in the Tokyo RCC cohort. HR_(low MTB score)_ = 3.8, Log-rank *p* value = 0.004.

To further explore the interconnections among gene clusters and MTB signature genes, we used enrichment map which is a functional grouping network diagram where each node represents a KEGG pathway (Figures 4(a) and 4(b)). And to collect quantitative pointers of the metabolic aspect in each case, we performed PCA to calculate the MTB scores, which were defined as the subtracting of the first principal component scores between MTB signature gene A and MTB signature gene B (Table S3). To generate the optimal cut-off value of the MTB scores, we used the survival R package to reach maximally selected rank statistics [47], which then categorized cases in the TCGA cohort into high or low MTB score groups. The allocation of patients among gene clusters, MTB score subgroups, and clinical characteristics is summarized in Figure 4(c) and Table 2. We then examined the differences in immune action and tolerance status between high and low MTB score groups where CD274, CTLA4, HAVCR2, IDO1, LAG3, and PDCD1 were chosen as immune check points and CD8A, CXCL10, CXCL9, GZMA, GZMB, PRF1, TBX2, and TNF as antitumor activity-related genes [48, 49]. We observed that the majority of the immune checkpoints and antitumor activity-related genes except CTLA4, LAG3, PDCD1, GZMB, and TNF were highly expressed in the high MTB group, as determined by the Wilcoxon test (Figure 4(d)).

Subsequently, we analyzed the prognostic value of the MTB score in the TCGA cohort, CTPAC-3, and Tokyo cohorts independently. Via K-M survival analysis, we observed that cases in the high MTB score group possessed significantly better OS in all three cohorts determined by the log-rank test (TCGA: *p* < 0.001, CTPAC-3: *p* < 0.001, Tokyo: *p* = 0.004, Figures 4(e)–4(g)). In comparison to earlier molecular subtype classifications [50, 51], we found that the high MTB score group mainly coincided with TCGA cluster I and ccA subtype which possessed better clinical outcomes whereas the low MTB score group was more in line with TCGA cluster III and ccB subtype which signified poor prognosis (Figure S3A).

### 3.4. The Association amid the MTB Scores and Somatic Mutations

**Figure 5:**
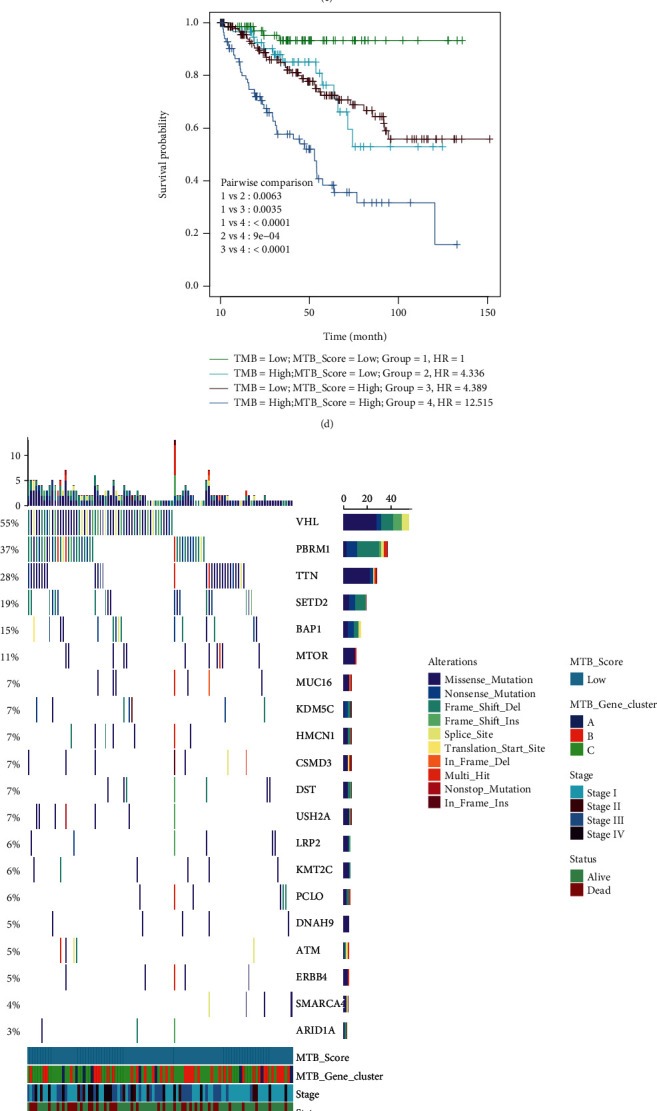
The association between the MTB score and somatic mutation. (a) Variation of the MTB score among MTB gene clusters (Kruskal-Wallis test *p* < 0.001). (b) Scatterplots describing the positive association in the MTB scores and TMB in the TCGA set (Spearman correlation *R* = 0.12, *p* = 0.022). (c) Survival analysis for patients with high and low TMB in the TCGA RCC cohort. HR_(high TMB)_ = 2.8, Log-rank *p* < 0.001. (d) Pairwise survival analysis of four groups (group 1: low TMB and low MTB score, group 2: high TMB and low MTB score, group 3: low TMB and high MTB score, group 4: high TMB and high MTB score). HR was generated by the Cox regression was group 1 as the base. ((e) and (f)) The oncoPrint plot was created with low MTB scores on the left (red) and high MTB scores on the right (red). Individual cases are depicted in the column.

Tumor mutational burden (TMB) has been viewed as a reliable indicator for immunotherapy response and OS in several solid cancers, and patients with high TMB tend to have better ICI treatment response [52–54]. However, in ccRCC, TMB seems unimpactful for immunotherapy response [55, 56]. And Zhang et al. found that higher TMB levels were associated with poor OS, conferring higher tumor grades and clinical stages in ccRCC patients [57]. Regarding the prognostic indications of TMB, we attempted to investigate the correlation between the TMB and the MTB scores to illustrate the genetic characteristics of each MTB subgroup. As displayed in Figures 5(a) and 5(b), cases in the different MTB gene clusters revealed various MTB scores (Kruskal—Wallis test, *p* < 0.001), and Spearman correlation analysis showed a positive correlation between the MTB scores and TMB (coefficient: *R* = 0.12, *p* = 0.022). To explore the prognostic value of TMB in RCC, we classified 365 TCGA RCC patients into high TMB and low TMB groups using the previous mentioned method [47]. We observed that patients with low TMB exhibited a more satisfying OS than those with high TMB group (log-rank *p* value < 0.001; Figure 5(c)). To consider the predictive value of TMB and the MTB score together, we next stratified cases in the TCGA cohort into four groups (group 1: low TMB and low MTB score; group 2: high TMB and low MTB score; group 3: low TMB and high MTB score; group 4: high TMB and high MTB score). And the Cox regression was applied to quantify the hazard ratio (HR). Pairwise survival analysis revealed that the prognostic value of the MTB score was not intervened by TMB status of individuals (group 1, HR = 1 versus group 2, HR = 4.336, *p* = 0.0063; group 3, HR = 4.389 versus group 4, HR = 12.515, *p* < 0.0001). Although stratified by different TMB groups, the high MTB score group held better OS than low MTB score group and the predictive value remained stable. In summary, these findings suggest that the MTB score can serve as a robust prognostic indicator independent of TMB.

In addition, we studied the occurrence of somatic mutations in RCC driver genes within the high and low MTB groups. The top 20 driver genes with the largest mutational frequency were acquired from the maftools [41] and analyzed (Figures 5(e) and 5(f)). Analysis of the nonsynonymous mutation exposed that the mutational frequency of gene PBRM1and TTN was significantly different between the high and low MTB score groups (Pearson's Chi-squared test; Fisher's exact test; Table 3. These results might render different approaches for studying the mechanism of tumor metabolic structure and gene mutation in ICI therapy.

### 3.5. Distinct Sensitivity to Immunotherapy and Targeted Therapies for MTB Subclasses

**Figure 6:**
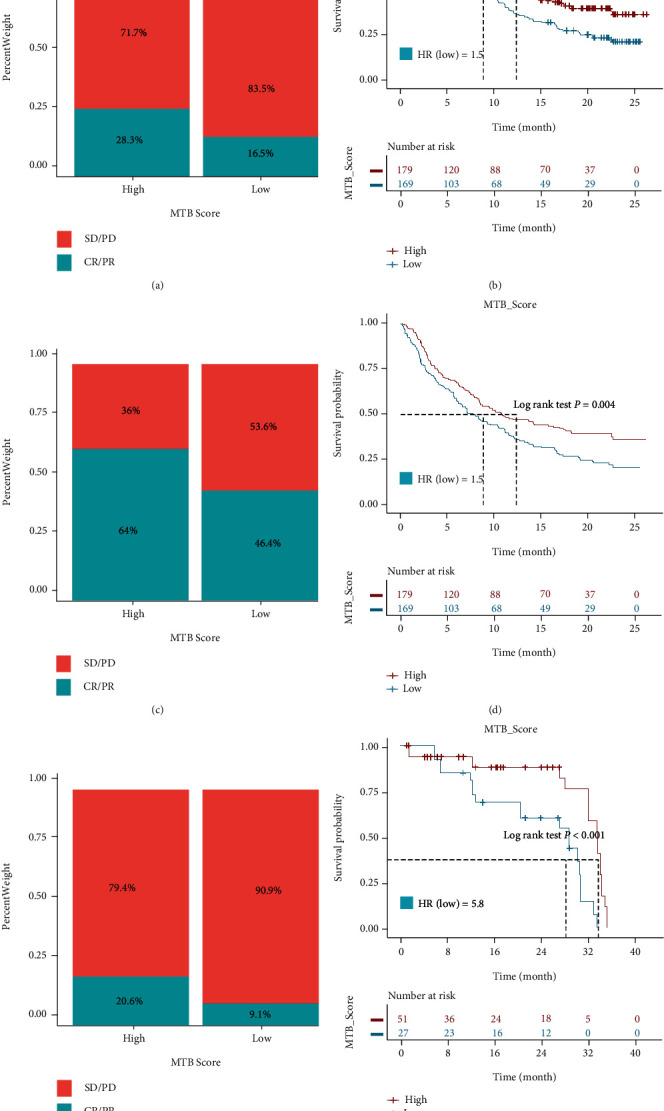
Evaluating the value of MTB scores in targeted therapy and immunotherapy. (a) Distribution of clinical response (CR: complete response; PR: partial response; and SD: stable disease; PD: progressive disease) to TKI therapy in high or low MTB score groups in the E-MTAB-3267 cohort. (b) Survival analysis for patients with high and low MTB score in the E-MTAB-3267 RCC cohort. HR_(low MTB score)_ = 3.6, Log-rank *p* value < 0.001. (c) Distribution of clinical response to anti-PD-L1 therapy in high or low MTB score groups in the IMvigor210 cohort. (d) Survival analysis for patients with high and low MTB score in the IMvigor210 cohort. HR_(low MTB score)_ = 1.5, Log-rank *p* = 0.004. (e) Distribution of clinical response to anti-PD-1 therapy in high or low MTB score groups in the E-MTAB-3218 RCC cohort. (f) Survival analysis for patients with high and low MTB score in the E-MTAB-3218 RCC cohort. HR_(low MTB score)_ = 5.8, Log-rank *p* value < 0.001.

TKI targeted therapy and ICI therapy are more and more becoming mainstream treatments for RCC patients who are naive to initial therapy. Still, the selection of suitable candidates remained intractable trouble because of the low effective rate [23, 58]. Thus, to explore the role of the MTB score in therapeutic benefit, we analyzed three independent cohorts receiving various therapies including E-MTAB-3267 treated with a TKI agent (sunitinib), IMvigor210 cohort treated with anti-PD-L1 agent (atezolizumab), and E-MTAB-3218 treated with anti-PD-1 agent (nivolumab). All cases were allocated into high or low MTB scores applying previously mentioned MSRS method independently. Distinctly, we found patients acquiring high MTB scores better outlived patients with low MTB scores in the TKI treated cohort (log-rank *p* value < 0.001; Figure 6(b)). And the treatment response occupation of TKI therapy was higher in the high MTB score class than in the low MTB class (Figure 6(a)). Similar results were obtained in the IMvigor210 cohort (log-rank *p* value = 0.004; Figures 6(c) and 6(d)) and were further validated in the E-MTAB-3218 cohort (Log-rank *p* value < 0.001; Figures 6(e) and 6(f)). Interestingly, in the E-MTAB-3218 cohort, we found the MTB score remained stable between pre- and post-ICI treatment (Wilcoxon *p* = 0.85; Figure S3B and S3C). To investigate its value in other TKI agents, we quantified the ridge regression model based on the GDSC cell line data and estimated the IC50 for each individual in the TCGA RCC cohort. The results, as shown in Figure S4 A-C, indicated that the low MTB score class showed higher sensitivity than the high MTB score class in sorafenib (*p* < 0.001), but not in pazopanib (*p* = 0.999), or axitinib (*p* = 0.757). These findings collectively suggest that the MTB score can serve as a novel prognostic symbol and indicator of immunotherapy response as well as some TKI therapy response.

## 4. Discussion

Immunotherapy has proven to be a powerful tool in eradicating human cancers and has remodeled the treatment paradigm of advanced renal cancer as well as earlier strategies, including antiangiogenic agents like sunitinib, sorafenib, pazopanib, and axitinib. Therefore, in the past few years, several immune checkpoint inhibitors (ICI) have been approved by the US Food Drug Administration and the European Medicines Agency for the treatment of mRCC, including nivoliumab as a single agent or in combination with ipilimumab, pembrolizumab in combination with axitinib, and avelumab in combination with axitinib [22, 59–61]. Though immunotherapy has benefited numerous RCC patients, a vast majority still suffer from disease progression. Hence, screening of patients, predicting the efficacy of targeted therapy, and guiding clinical drug use are becoming increasingly important. In the current study, we built a method to quantify the complete tumor metabolism milieu in RCC. Our study's results showed that the MTB score is a valuable prognostic biomarker as well as a predictive indicator for estimating immunotherapy and targeted therapy effectiveness.

There is a growing body of literature that recognizes the dysfunction of metabolic pathways within tumors boosted cancer cell proliferation and affected drug treatment [62]. It is now well established that RCC is driven by dysregulated metabolism due to the highly mutated genes that manage various metabolic characteristics, including mutated VHL in the hypoxia pathway, mutation of MTOR and PTEN in PI3K–Akt–mTOR axis, and genes such as FH and SDH respond to nutrient stimulation [63]. These aberrant metabolism-related pathways do not only nurture tumor proliferation and orchestrating TME but also act on drug treatment response as well [16]. Prior studies from Zhao et al. have noted the importance of fatty acid metabolism in ccRCC progress and focused on certain multiple fatty acid metabolic enzymes including CPT1A, HADHA, HADHB, and ACAT1 while no extra multiomics data or clinical treatment cohorts were incorporated [12]. A strong relationship between metabolites (succinate, beta-alanine, purines, glucose, and myo-inositol) and clinical outcomes in ccRCC has been reported in previously mentioned literature which integrated transcriptomic and metabolomic analyses while their predictive value in drug treatment remained unexplored [13]. The present study was designed to explore the broad picture of metabolic pathways of ccRCC patients and discover molecular subtypes which could serve as robust prognostic biomarkers and treatment indicators as well. We investigated the metabolic pathways in a metadata set of 729 RCC individuals and classified them into three separate MTB subtypes. Among the four ICI-resistance-associated pathways, MTB cluster C2 with unfavored prognosis demonstrated elevated score of hypoxia and Wnt/Beta-catenin signalings which were consistent with existing studies that HIF-2*α* played a key role in tumor hypoxia environment and Wnt/Beta-catenin signalings produced protumourigenic activities [64, 65]. And gene cluster A with better OS manifested declined score of hypoxia and Wnt/Beta-catenin signaling which may be more likely to benefit from ICI therapy.

RCC holds one of the most immune cell-infiltrated tumors among all solid cancers, and hallmarks of the TME massively modify cancer biology and may influence responses to various therapies [66]. And cancer metabolism closely interacts with immune activities in the TME [15]. Our report showed that MTB cluster C2 exhibited the highest immune score and stromal score which may contribute to its poor outcomes, and this result is in line with previous findings [67, 68]. Gene cluster C displayed decreased myeloid dendritic cell enrichment, which was associated with previously reported “immune exhausted phenotype” [69]. And for further study, we collected potential biomarkers and built the MTB score to calculate the metabolic pattern of individuals using the Boruta algorithm. And we found immune checkpoints and antitumor activity-related genes were lower in low MTB score groups which may contribute to the unsatisfactory OS of patients and various MTB scores and expression patterns of immune checkpoints, and their function-related genes among RCC subclasses showed that the probability of responding to ICI therapy calls for further investigation. Moreover, recent clinical trials have recognized a connection between the genetic variations and clinical response for immunotherapy [70]. Here, we found the mutation frequency PBRM1 was significantly higher in the high MTB score group which had been proven to be a predictive biomarker for ICI treatment in ccRCC [71]. TTN, which has highly mutated in the low MTB score group, was discovered to be associated with prognosis and immunotherapy efficacy in multiple cancers [72]. Additionally, we discovered that TMB was significantly correlated with the MTB score (*r* = 0.1227). The pairwise survival analysis disclosed that the predictive value of MTB scores was free from TMB. In this investigation, we assumed that the comprehensive quantification of metabolic profiles and metabolism-related genes in RCC patients would be an innovative strategy for choosing optimal therapy for a specific patient.

Likewise, fluctuations in metabolic status exert major effects on immunotherapy among several solid cancer types. With respect to melanoma, Harel et al. discovered lipid metabolism can enhance tumor antigen presentation and thus improve response to ICI therapy [73]. Interestingly, a metabolic-tumor-stroma score (MeTS) system has recently been proposed, which may guide cancer immunotherapy across cancer types. Based on varied cellular and metabolic heterogeneity, cancers including breast cancer, pancreatic cancer, and colon cancer are classified into four subtypes although the sample size is quite limited [74]. Further, through metabolomic profiling, Gong and her team were able to classify triple-negative breast cancer patients into three subgroups which showed different sensitivity to current therapy [75]. These findings further proved that the intrinsic metabolic status of tumors could be utilized for therapeutic guidance.

The IMvigor210, E-MTAB-3218, and E-MTAB-3267 datasets were assessed for patients receiving ICI therapy and anti-VEGF therapy. We noticed that the MTB score was significantly raised in patients who responded to ICI therapy and anti-VEGF therapy and proved its predictive utility. Overall, this implies that ICI therapy and anti-VEGF agents might be helpful in cases with high MTB scores. Therefore, deepening the understanding of cancer immunotherapy, perfecting existing biomarkers, and developing new tumor markers are important development directions for immunotherapy in the future. We look forward to further research or optimization of different combination schemes through the exploration of advantageous populations and biomarkers.

## 5. Conclusions

In conclusion, we comprehensively investigated the metabolic aspects of ccRCC, presenting a clear understanding of the immune response and metabolic variations in the TCGA RCC cohort. Variations in MTB scores were associated with cancer heterogeneity, treatment regimen, and clinical outcomes. Hence, the evaluation of tumor MTB scores conducted by our research has critical clinical indications. In addition, the results will help in the classification of fitting applicants for personalized medical treatment and improve the clinical benefits of patients with ccRCC.

## Figures and Tables

**Table 1:** Clinical characteristics of three ccRCC cohorts (TCGA, CTPAC-3, and Tokyo).

Characteristic	TCGA, *N* = 530^1^	CTPAC-3, *N* = 110^1^	Tokyo, *N* = 101^1^
Age	61 (52, 70)	61 (53, 70)	64 (56, 72)
Gender			
Female	186 (35%)	30 (27%)	24 (24%)
Male	344 (65%)	80 (73%)	77 (76%)
Stage			
Stage I	266 (50%)	52 (47%)	66 (65%)
Stage II	57 (11%)	13 (12%)	10 (9.9%)
Stage III	123 (23%)	33 (30%)	13 (13%)
Stage IV	81 (15%)	12 (11%)	12 (12%)
Unknown	3 (0.6%)	0 (0%)	0 (0%)
Vital status			
Alive	357 (67%)	96 (87%)	78 (77%)
Dead	173 (33%)	14 (13%)	23 (23%)
Follow-up time	40 (19, 64)	23 (14, 36)	51 (34, 81)
Unknown	2	10	0

^1^Median (IQR); *n* (%).

**Table 2:** Clinical characteristics between MTB score subgroups (TCGA, CTPAC-3, and Tokyo).

Characteristic	MTB score, high, *N* = 421^1^	MTB score low, *N* = 320^1^
Age	61 (52, 71)	61 (53, 70)
Gender		
Female	155 (37%)	85 (27%)
Male	266 (63%)	235 (73%)
Stage		
Stage I	245 (58%)	139 (43%)
Stage II	47 (11%)	33 (10%)
Stage III	88 (21%)	81 (25%)
Stage IV	40 (9.5%)	65 (20%)
Unknown	1 (0.2%)	2 (0.6%)
Vital status		
Alive	346 (82%)	185 (58%)
Dead	75 (18%)	135 (42%)
Follow-up time	40 (23, 66)	34 (16, 58)
Unknown	6	6
Project		
CTPAC-3	72 (17%)	38 (12%)
TCGA	302 (72%)	228 (71%)
Tokyo	47 (11%)	54 (17%)

^1^Median (IQR); *n* (%).

**Table 3:** Connection between MTB scores and somatic variants.

Characteristic	High, *N* = 184^1^	Low, *N* = 100^1^	*p* value^2^
VHL	111 (60%)	55 (55%)	0.4
PBRM1	103 (56%)	37 (37%)	0.002
TTN	31 (17%)	28 (28%)	0.027
SETD2	23 (12%)	19 (19%)	0.14
BAP1	20 (11%)	15 (15%)	0.3
MTOR	11 (6.0%)	11 (11%)	0.13
MUC16	15 (8.2%)	7 (7.0%)	0.7
KDM5C	13 (7.1%)	7 (7.0%)	>0.9
HMCN1	11 (6.0%)	7 (7.0%)	0.7
DNAH9	12 (6.5%)	5 (5.0%)	0.6

^1^ *n* (%). ^2^Pearson's Chi-squared test; Fisher's exact test.

## Data Availability

All datasets can be downloaded from Cancer Genome Atlas (TCGA, http://cancergehttp://nome.nih.gov/), the Array Express Database (AED, https://www.ebi.ac.uk/arrayexpress/), and http://research-pub.gene.com/IMvigor210CoreBiologies. Details are listed in the Materials and Methods part. And the R codes involving major steps were uploaded at https://github.com/yao50985098/MTB-Subtypes.

## Supplementary Materials

Supplementary Materials Supplementary Materials Figure S1-S4 with corresponding legends (.docx file) and Table S1-S3 (.pdf files) were uploaded in the Supplemental Files.
