# Supplementary material for: Comprehensive Characterization of Metabolism-Associated Subtypes of Renal Cell Carcinoma to Aid Clinical Therapy
Source: Oxid Med Cell Longev. 2022 Feb 27;2022:9039732. doi: 10.1155/2022/9039732 (PMC8898770; doi:10.1155/2022/9039732)
Supplement: Supplementary Materials — Figure S1-S4 with corresponding legends (.docx file) and Table S1-S3 (.pdf files) were uploaded in the Supplemental Files. [file 9039732.f1.zip › Supplementary Figure (1).docx]

**Figure S1**. **(A)** Raw PCA plot for three independent RCC cohorts. **(B)** Customized PCA plot after removing batch effects via Combat function in sva package. **(C, D and E)** Consensus matrixes of combined RCC cohort for k = 2-4, demonstrating the stability utilizing 1000 repetitions of hierarchical clustering.

**Figure S2. (A, B and C)** Consensus matrixes of TCGA RCC cohort for k = 2-4, demonstrating the stability utilizing 1000 repetitions of hierarchical clustering. **(D)** Venn plot to demonstrate the fraction of DEGs amid the three MTB clusters.


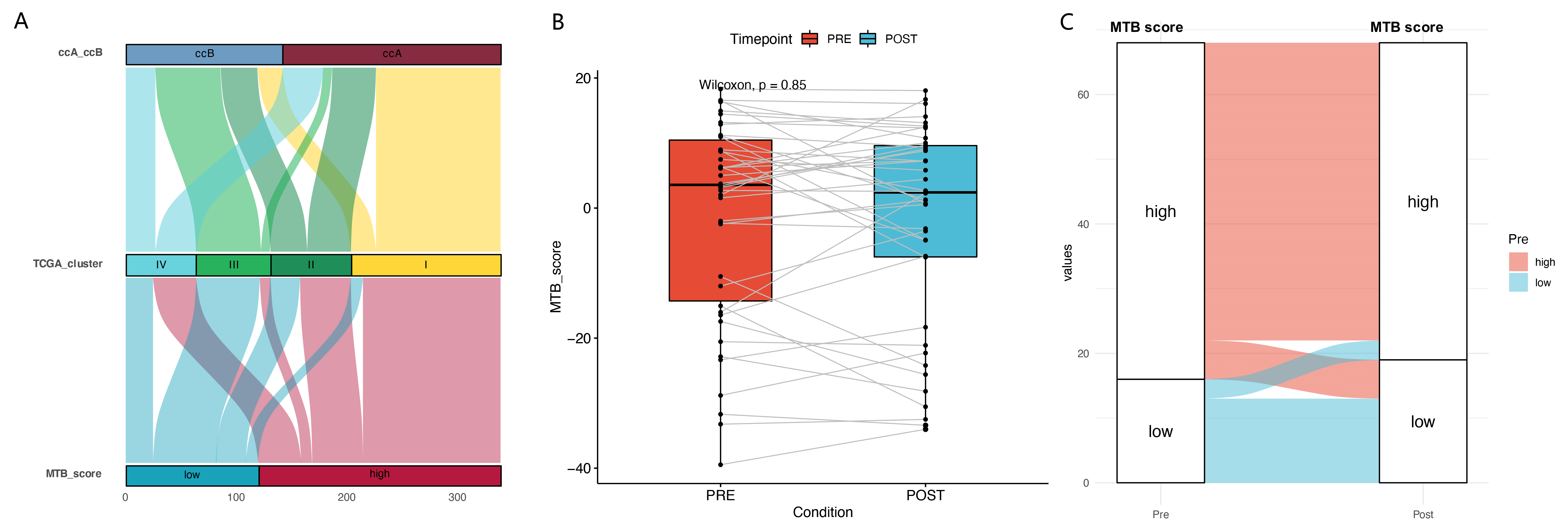


**Figure S3.** **(A)** The relation of MTB score subgroups among the TCGA clusters and ccA/ccB subgroups. (**B)** MTB scores between pre- and post-ICI treatment of 44 RCC patients in the E-MTAB-3218 cohort. Wilcoxon p = 0.85. **(C)** Alluvial chart of MTB score division between pre- and post-ICI treatment.


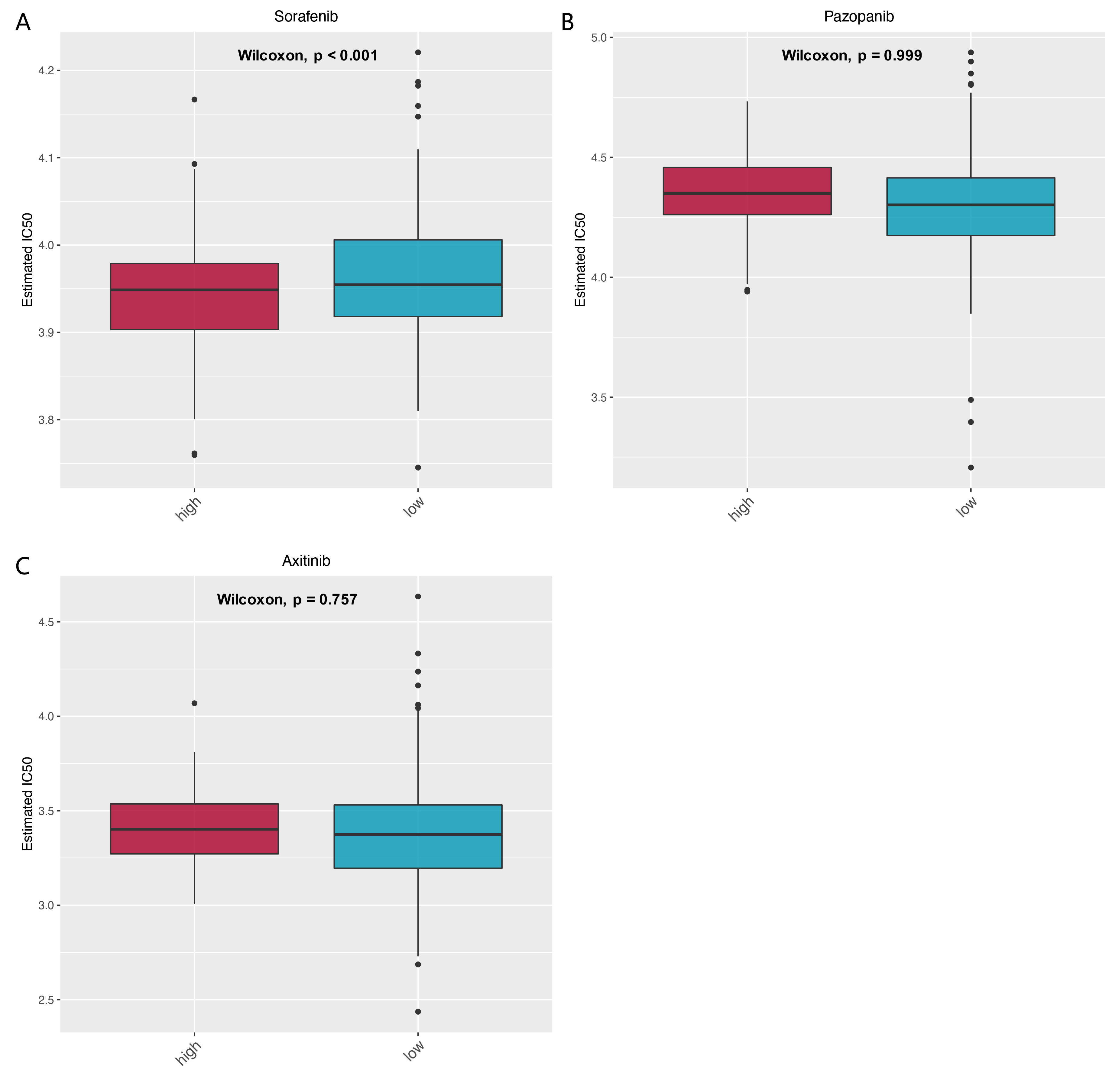


**Figure S4. (A, B and C)** Predicted IC50 of TKI agents including sorafenib, pazopanib, and axitinib to evaluate therapeutic response among MTB score subgroups.
