# Supplementary material for: Comprehensive Characterization of Metabolism-Associated Subtypes of Renal Cell Carcinoma to Aid Clinical Therapy
Source: Oxid Med Cell Longev. 2022 Feb 27;2022:9039732. doi: 10.1155/2022/9039732 (PMC8898770; doi:10.1155/2022/9039732)
Supplement: Supplementary Materials — Figure S1-S4 with corresponding legends (.docx file) and Table S1-S3 (.pdf files) were uploaded in the Supplemental Files. [file 9039732.f1.zip › Table S1 (1).pdf]

gene	r	p	direct
SLPI	0.66930715	3.90E-70	A
SLC4A3	0.66657572	2.23E-69	A
TRNP1	0.66257654	2.77E-68	A
GFPT2	0.65572687	1.90E-66	A
GPRC5A	0.6527254	1.17E-65	A
UCHL1	0.61918084	2.05E-57	A
TMEM158	0.60619809	1.74E-54	A
MOCOS	0.60146039	1.89E-53	A
SEMA3C	0.59428848	6.49E-52	A
IGF2BP2	0.59066274	3.75E-51	A
MAGED4B	0.58498945	5.58E-50	A
BDKRB1	0.58003806	5.64E-49	A
CPXM1	0.57972897	6.50E-49	A
LEF1	0.57172348	2.51E-47	A
IGDCC4	0.57110836	3.31E-47	A
EFNA5	0.56993907	5.60E-47	A
MFI2	0.56950317	6.80E-47	A
RARRES1	0.56632296	2.79E-46	A
HOXB9	0.56349625	9.69E-46	A
DMRT3	0.56305178	1.18E-45	A
SERPINA3	0.56080041	3.14E-45	A
ITPKA	0.56063216	3.37E-45	A
ADAM12	0.56046174	3.63E-45	A
PPP1R1A	0.5600687	4.30E-45	A
CLMP	0.55975278	4.93E-45	A
ROR2	0.55872454	7.69E-45	A
PTGES	0.55207983	1.30E-43	A
KCNG1	0.55082551	2.21E-43	A
PKP3	0.5502997	2.75E-43	A
SRPX2	0.54704954	1.06E-42	A
KCNS1	0.54695095	1.11E-42	A
RAB3B	0.5454443	2.06E-42	A
CDCP1	0.54446473	3.09E-42	A
PAEP	0.54444819	3.11E-42	A
10-Mar	0.54373872	4.16E-42	A
SYT8	0.54302101	5.58E-42	A
SLC12A8	0.54249878	6.90E-42	A
SPTBN2	0.53998363	1.92E-41	A
C10orf90	0.53973379	2.12E-41	A
MAT1A	0.53804509	4.19E-41	A
PROM2	0.53585596	1.01E-40	A
SLC38A5	0.53493922	1.45E-40	A
LAMB3	0.53430494	1.86E-40	A
STEAP3	0.53409045	2.03E-40	A
GAD1	0.53286875	3.29E-40	A

SAA2	0.53212258	4.41E-40	A
ARL14	0.53011855	9.69E-40	A
PDGFRL	0.52635738	4.18E-39	A
MOXD1	0.52507196	6.86E-39	A
LOXL1	0.52448442	8.60E-39	A
CILP2	0.52383728	1.10E-38	A
PODNL1	0.52309816	1.46E-38	A
SAA1	0.52174458	2.45E-38	A
TFAP2A	0.51992305	4.89E-38	A
KLK13	0.51874782	7.62E-38	A
PTPRH	0.51767228	1.14E-37	A
SIX4	0.51630891	1.90E-37	A
ACTBL2	0.51444045	3.81E-37	A
PI3	0.51271273	7.23E-37	A
FGF7	0.51247334	7.90E-37	A
ERC2	0.51130895	1.21E-36	A
CCDC80	0.51075237	1.49E-36	A
TUBB3	0.51026867	1.78E-36	A
STMN2	0.50852561	3.36E-36	A
CHRDL2	0.50796444	4.12E-36	A
COL11A1	0.50789724	4.22E-36	A
IGFBP1	0.50778063	4.40E-36	A
SERPINA5	0.50658698	6.78E-36	A
PVRL4	0.50612822	8.01E-36	A
F3	0.50565319	9.51E-36	A
CDH3	0.50483465	1.28E-35	A
CREB3L1	0.50428598	1.55E-35	A
CPEB1	0.50399414	1.73E-35	A
STRA6	0.502814	2.63E-35	A
LUM	0.50208275	3.42E-35	A
FAM101A	0.50126243	4.58E-35	A
CPA4	0.50107517	4.89E-35	A
KRT15	0.49881595	1.09E-34	A
ONECUT2	0.49744286	1.76E-34	A
ADAMTS14	0.49539288	3.61E-34	A
KCNK15	0.49529299	3.74E-34	A
LYPD1	0.49511072	3.99E-34	A
FNDC4	0.4934446	7.11E-34	A
TMC5	0.49337132	7.30E-34	A
PANX2	0.49093781	1.69E-33	A
APCDD1L	0.48976934	2.52E-33	A
SYNGR3	0.48705164	6.36E-33	A
PLTP	0.48662077	7.37E-33	A
C14orf132	0.48651795	7.63E-33	A
WBSCR17	0.48649183	7.69E-33	A
REEP2	0.48500621	1.27E-32	A

WFDC5	0.47938203	8.30E-32	A
C20orf141	0.47796216	1.33E-31	A
KRT19	0.47747271	1.56E-31	A
PSAT1	0.47736337	1.62E-31	A
GCKR	0.47697885	1.83E-31	A
CCNA1	0.47350828	5.69E-31	A
GREM1	0.47038112	1.56E-30	A
SPOCD1	0.47025637	1.62E-30	A
KLF17	0.46977591	1.89E-30	A
DNER	0.46949701	2.07E-30	A
COL10A1	0.46935148	2.17E-30	A
PPP2R2C	0.46906372	2.38E-30	A
WISP2	0.4667144	5.02E-30	A
UCN2	0.46642761	5.50E-30	A
BMPR1B	0.46618291	5.95E-30	A
LRRC15	0.46573437	6.85E-30	A
CCNO	0.46443054	1.03E-29	A
COL7A1	0.46327422	1.49E-29	A
CAPN8	0.46288624	1.68E-29	A
SPEG	0.46281958	1.71E-29	A
MFAP2	0.46152551	2.57E-29	A
XK	0.46047723	3.56E-29	A
LMO1	0.46040472	3.64E-29	A
MAGEL2	0.46039831	3.65E-29	A
ETV4	0.46014231	3.95E-29	A
TFAP2C	0.45984143	4.34E-29	A
LIPH	0.45909288	5.47E-29	A
FAP	0.45898073	5.66E-29	A
TCN1	0.45891058	5.78E-29	A
VSTM2L	0.45734359	9.38E-29	A
ZNF365	0.45717878	9.86E-29	A
GYG2	0.45558464	1.61E-28	A
GSDMC	0.45463499	2.15E-28	A
SH3GL3	0.45359163	2.95E-28	A
HTR3A	0.4535297	3.01E-28	A
GJB3	0.45321269	3.31E-28	A
MMP12	0.45186185	4.98E-28	A
PTX3	0.44976155	9.38E-28	A
TGM5	0.44886552	1.23E-27	A
CRABP1	0.44804175	1.57E-27	A
TGFBI	0.44768982	1.74E-27	A
CCL11	0.44765998	1.76E-27	A
WNT2	0.44762196	1.78E-27	A
SBSN	0.4469039	2.20E-27	A
FRMD5	0.44679701	2.27E-27	A
PDGFRA	0.44612026	2.78E-27	A

UBE2C	0.44494028	3.93E-27	A
DCN	0.44487948	4.01E-27	A
KLK10	0.44441793	4.59E-27	A
MMP13	0.44409847	5.04E-27	A
SLC22A31	0.44348895	6.03E-27	A
NTNG1	0.44298248	7.00E-27	A
SPINK1	0.44228264	8.59E-27	A
TNNT1	0.44169496	1.02E-26	A
PCBP3-OT1	0.44156803	1.06E-26	A
SLC7A5	0.43912195	2.16E-26	A
HMGA2	0.43873014	2.41E-26	A
IL6	0.43839926	2.66E-26	A
TMEM61	0.43759189	3.35E-26	A
NMU	0.43724725	3.70E-26	A
FLNC	0.4367141	4.32E-26	A
HP	0.4363151	4.84E-26	A
SERPINA4	0.43529184	6.49E-26	A
LINC00460	0.43504691	6.96E-26	A
EPB41L4B	0.43481492	7.43E-26	A
PI15	0.43450957	8.11E-26	A
GPR87	0.43414957	8.98E-26	A
PYGO1	0.43338758	1.12E-25	A
SAA4	0.43327502	1.15E-25	A
ZFHX4	0.4329912	1.25E-25	A
PYCR1	0.43284426	1.30E-25	A
TFCP2L1	0.43110047	2.13E-25	A
HSD11B1	0.43092604	2.24E-25	A
DUSP9	0.43022145	2.73E-25	A
CTNNA2	0.42878083	4.09E-25	A
PLEKHG4B	0.42863009	4.26E-25	A
KLK1	0.42721228	6.33E-25	A
NALCN	0.42539805	1.05E-24	A
PTGER1	0.42529355	1.08E-24	A
CYP11A1	0.42449477	1.34E-24	A
SMPX	0.42385983	1.60E-24	A
KLC3	0.42296573	2.05E-24	A
NIPAL4	0.4223391	2.43E-24	A
MDK	0.42217204	2.54E-24	A
WFDC10B	0.41902979	5.98E-24	A
PLA2G2A	0.41767497	8.62E-24	A
ISLR	0.41698759	1.04E-23	A
IGF2BP3	0.41684055	1.08E-23	A
COMP	0.41596371	1.37E-23	A
G0S2	0.41579057	1.43E-23	A
MMP9	0.41449401	2.02E-23	A
EHF	0.41442132	2.06E-23	A

ARL9	0.4115673	4.40E-23	A
ARSI	0.41127154	4.75E-23	A
WNT4	0.41001063	6.63E-23	A
GDF5	0.40968243	7.22E-23	A
PCP4	0.40928354	8.02E-23	A
SLN	0.40906512	8.49E-23	A
WNT7B	0.40851124	9.81E-23	A
CTHRC1	0.40798343	1.13E-22	A
KRT79	0.40556167	2.11E-22	A
CST2	0.40526529	2.28E-22	A
SCN3A	0.40472595	2.62E-22	A
ZPLD1	0.40344693	3.64E-22	A
ROS1	0.40331968	3.77E-22	A
EGFL6	0.40256816	4.57E-22	A
CYP19A1	0.40231528	4.87E-22	A
CFHR1	0.40133474	6.26E-22	A
XDH	0.40106435	6.70E-22	A
FGF9	0.40068179	7.39E-22	A
NKX6-1	0.39949093	1.00E-21	A
SFRP2	0.39842185	1.31E-21	A
MKX	0.39782353	1.52E-21	A
KCNK17	0.39740905	1.69E-21	A
DYDC2	0.39644671	2.15E-21	A
CST5	0.39478973	3.26E-21	A
CDH15	0.39306028	5.01E-21	A
CHRDL1	0.39264815	5.55E-21	A
WFDC12	0.39259585	5.62E-21	A
COL1A1	0.39219125	6.22E-21	A
IBSP	0.392034	6.46E-21	A
AGR2	0.39192101	6.65E-21	A
EMILIN1	0.39183841	6.78E-21	A
RHCG	0.39124463	7.85E-21	A
TGM1	0.39065321	9.08E-21	A
NKX2-8	0.39004187	1.06E-20	A
ZIC5	0.39003937	1.06E-20	A
SP5	0.38969514	1.15E-20	A
DMBX1	0.38936554	1.25E-20	A
THBS2	0.38914797	1.31E-20	A
INSRR	0.38905564	1.34E-20	A
RYR2	0.3878405	1.81E-20	A
PYY	0.38756817	1.93E-20	A
IGSF5	0.38752332	1.95E-20	A
ZP1	0.38719008	2.12E-20	A
FXYD3	0.38714645	2.14E-20	A
RHBG	0.38713192	2.15E-20	A
VWDE	0.38692373	2.26E-20	A

EFEMP1	0.38607714	2.77E-20	A
HPR	0.38550241	3.19E-20	A
CLCNKA	0.38401165	4.57E-20	A
FLJ26245	0.3838342	4.76E-20	A
EPHA10	0.38368892	4.93E-20	A
ZIC2	0.38351304	5.15E-20	A
PRSS3	0.38291552	5.94E-20	A
COL22A1	0.38129496	8.75E-20	A
GUCY2D	0.37954303	1.33E-19	A
DRP2	0.37902166	1.50E-19	A
L1CAM	0.37902054	1.50E-19	A
COL16A1	0.37833279	1.76E-19	A
FOXE1	0.37717095	2.32E-19	A
CCBE1	0.3768681	2.49E-19	A
KRT78	0.37685404	2.50E-19	A
ERN2	0.3759287	3.10E-19	A
SCG5	0.37566082	3.30E-19	A
TMEM74	0.37498452	3.87E-19	A
FAM83B	0.37440916	4.42E-19	A
FETUB	0.37360607	5.33E-19	A
KLK4	0.37336885	5.63E-19	A
HRASLS	0.3715446	8.58E-19	A
GSG1	0.37028606	1.15E-18	A
FGL1	0.36888315	1.58E-18	A
TNNI3	0.36757136	2.13E-18	A
EN1	0.36710432	2.37E-18	A
CHAT	0.36512603	3.70E-18	A
COL5A1	0.36491073	3.88E-18	A
TMPRSS7	0.36408378	4.67E-18	A
PPP1R1B	0.3640816	4.68E-18	A
IGFL2	0.36353215	5.29E-18	A
PODN	0.36343866	5.40E-18	A
PADI3	0.36258329	6.54E-18	A
CPZ	0.3623445	6.89E-18	A
CCL26	0.36234064	6.90E-18	A
LBP	0.36165729	8.03E-18	A
HAS2-AS1	0.36015945	1.12E-17	A
HOXB13	0.35996185	1.17E-17	A
LHFPL5	0.35992589	1.18E-17	A
PCSK9	0.35932368	1.35E-17	A
COX6B2	0.35827738	1.70E-17	A
COL6A3	0.35669967	2.40E-17	A
KRT17	0.35632446	2.60E-17	A
SLC7A4	0.35550733	3.11E-17	A
IGFN1	0.35543118	3.16E-17	A
RPSAP52	0.35487053	3.57E-17	A

PVALB	0.35402513	4.29E-17	A
GRM4	0.35299594	5.36E-17	A
EPN3	0.35237207	6.13E-17	A
LHX1	0.35188676	6.80E-17	A
PLA2G4F	0.35134823	7.63E-17	A
NR0B1	0.35068252	8.80E-17	A
DKKL1	0.35010081	9.97E-17	A
IL11	0.34986569	1.05E-16	A
WFDC13	0.3497967	1.06E-16	A
F2	0.34917888	1.21E-16	A
LCN2	0.3475819	1.70E-16	A
MFAP4	0.3462918	2.23E-16	A
IL20RB	0.34537468	2.71E-16	A
MFSD2A	0.34523204	2.79E-16	A
RGS20	0.3444293	3.30E-16	A
FBN3	0.34403124	3.59E-16	A
KRT16	0.34354443	3.97E-16	A
B4GALNT1	0.34264584	4.79E-16	A
S100A5	0.34260717	4.83E-16	A
SLC18A3	0.34238931	5.05E-16	A
SPOCK1	0.33971281	8.79E-16	A
ADAM33	0.3392444	9.67E-16	A
PDPN	0.33919529	9.77E-16	A
CHST4	0.33913629	9.89E-16	A
SSC5D	0.33809533	1.22E-15	A
RBM11	0.33761816	1.35E-15	A
COL1A2	0.33754263	1.37E-15	A
PRKCG	0.33738542	1.42E-15	A
CCL21	0.33712742	1.49E-15	A
CCL13	0.33603397	1.86E-15	A
C4BPA	0.33514967	2.23E-15	A
SFTA2	0.33367908	3.00E-15	A
C19orf80	0.33343779	3.15E-15	A
ODAM	0.33333821	3.21E-15	A
CRABP2	0.33316264	3.33E-15	A
GRHL2	0.33301047	3.43E-15	A
LINC00323	0.33179446	4.37E-15	A
KRT39	0.32850666	8.40E-15	A
DMRT2	0.32771191	9.83E-15	A
LHX9	0.32711423	1.11E-14	A
MMP23B	0.32700195	1.13E-14	A
KLK8	0.32635535	1.28E-14	A
SFN	0.32609695	1.35E-14	A
STXBP5L	0.32524217	1.59E-14	A
CNGB1	0.32254428	2.69E-14	A
MIR31HG	0.32247772	2.73E-14	A

VSNL1	0.32221044	2.87E-14	A
NKX3-2	0.32135978	3.38E-14	A
CKMT1A	0.32033408	4.12E-14	A
INHA	0.32022752	4.20E-14	A
BFSP2	0.31957965	4.75E-14	A
CRHR1	0.31913126	5.18E-14	A
SNCB	0.31774179	6.75E-14	A
LRRC4C	0.31638973	8.71E-14	A
BARX1	0.31623791	8.96E-14	A
CDHR4	0.31568977	9.94E-14	A
B4GALNT4	0.31540905	1.05E-13	A
RCOR2	0.31508328	1.11E-13	A
SEMA3E	0.31505958	1.12E-13	A
PTGIS	0.31373467	1.43E-13	A
CDK5R2	0.31153442	2.16E-13	A
PRSS22	0.31109347	2.34E-13	A
GOLGA6L7F	0.30834698	3.88E-13	A
GPR78	0.30785893	4.25E-13	A
C6orf118	0.30705545	4.92E-13	A
PSCA	0.30537889	6.67E-13	A
FOXA1	0.30512788	6.98E-13	A
NPFFR2	0.30478317	7.42E-13	A
PITX1	0.30426624	8.15E-13	A
FGFBP1	0.30423485	8.20E-13	A
MRAP2	0.30377067	8.91E-13	A
GJB6	0.30246264	1.13E-12	A
ARHGAP40	0.30183659	1.26E-12	A
ARMC4	0.30019549	1.69E-12	A
GCGR	0.29919348	2.02E-12	A
ANKRD18B	0.29772442	2.61E-12	A
GNB3	0.29600796	3.53E-12	A
ELFN2	0.29520193	4.06E-12	A
FGA	0.2951457	4.10E-12	A
SULT4A1	0.29467065	4.45E-12	A
C6orf141	0.29378615	5.19E-12	A
CASP14	0.29280487	6.15E-12	A
KLK6	0.2897967	1.03E-11	A
GRP	0.28964546	1.06E-11	A
PSG5	0.28957665	1.07E-11	A
PRAME	0.28852584	1.28E-11	A
STAC2	0.28818784	1.35E-11	A
MMP3	0.28817425	1.36E-11	A
OTX1	0.28672847	1.73E-11	A
HSPB3	0.28458692	2.48E-11	A
IGFL3	0.28444087	2.54E-11	A
LIPI	0.28385802	2.80E-11	A

IGF2-AS	0.28352835	2.95E-11	A
FAM83E	0.28339122	3.02E-11	A
CHD5	0.2826955	3.39E-11	A
HAMP	0.28138759	4.21E-11	A
DKFZp434J0	0.27886834	6.36E-11	A
FAM19A3	0.27820937	7.08E-11	A
HAS1	0.27674592	8.98E-11	A
TMEM151A	0.2746916	1.25E-10	A
OVOL2	0.27451669	1.29E-10	A
SHD	0.26853294	3.32E-10	A
EEF1A2	0.26752218	3.88E-10	A
FAM83A	0.26694751	4.24E-10	A
FGG	0.26657381	4.50E-10	A
DCAF12L2	0.26641879	4.61E-10	A
HHATL	0.26419335	6.49E-10	A
LRFN5	0.26395695	6.73E-10	A
SLC6A20	0.26066354	1.11E-09	A
CCL7	0.25952061	1.32E-09	A
DUSP13	0.2581653	1.62E-09	A
CCIN	0.25727172	1.85E-09	A
AGR3	0.2569785	1.93E-09	A
CPA5	0.25507966	2.56E-09	A
FAM92B	0.25405795	2.98E-09	A
NPPB	0.25360936	3.18E-09	A
DMP1	0.25257795	3.70E-09	A
DUOXA1	0.25248935	3.75E-09	A
PITX2	0.2506223	4.92E-09	A
SLC30A10	0.25056774	4.96E-09	A
CST6	0.24954244	5.76E-09	A
TBX1	0.24569857	9.98E-09	A
PRRX2	0.24380217	1.30E-08	A
SLAMF9	0.24342234	1.38E-08	A
KRT13	0.24146302	1.81E-08	A
KLK11	0.23783096	2.99E-08	A
IGF2	0.23302029	5.74E-08	A
TFF1	0.23206097	6.53E-08	A
ELSPBP1	0.2297168	8.91E-08	A
IGSF23	0.22937746	9.32E-08	A
CXorf49B	0.22865737	1.02E-07	A
FGB	0.22292891	2.15E-07	A
AKNAD1	0.22107412	2.73E-07	A
NTF4	0.22032026	3.00E-07	A
DRD2	0.21853763	3.76E-07	A
CHGA	0.21542391	5.54E-07	A
LEMD1	0.21454578	6.17E-07	A
NKX2-5	0.21362591	6.91E-07	A

APOH	0.2093545	1.16E-06	A
IFNE	0.2076794	1.42E-06	A
REG3G	0.20503804	1.94E-06	A
DQX1	0.20327564	2.38E-06	A
GBX2	0.19897845	3.91E-06	A
LCN1	0.19638845	5.24E-06	A
ADAM7	0.19487843	6.21E-06	A
CPN2	0.19284691	7.78E-06	A
KC6	0.19138551	9.13E-06	A
KRT14	0.19130331	9.22E-06	A
MIA	0.18953482	1.12E-05	A
SEL1L2	0.18091969	2.79E-05	A
MIAT	0.18066709	2.87E-05	A
SALL4	0.18052869	2.91E-05	A
UNC93A	0.180276	2.98E-05	A
POM121L9P	0.17604669	4.60E-05	A
INHBE	0.17548243	4.87E-05	A
LINC00313	0.17473823	5.24E-05	A
CYP4F2	0.17355295	5.90E-05	A
TTTY2	0.1730554	6.20E-05	A
TAC3	0.16649813	0.00011767	A
KLK3	0.16644306	0.00011829	A
PRR20B	0.16397492	0.00014958	A
MUC19	0.15401369	0.00037292	A
SNORD3B-1	0.15245495	0.00042814	A
OBP2A	0.15025037	0.00051934	A
CSH1	0.14751889	0.00065735	A
SRPK3	0.14431783	0.00086206	A
SPANXA1	0.14366092	0.00091076	A
SLC26A10	0.14003793	0.00122822	A
GP1BB	0.12709721	0.00337891	A
SLC1A6	0.12619832	0.00361329	A
APOC3	0.12479047	0.00401012	A
HES7	0.11483947	0.00813725	A
HIST1H1D	0.11298134	0.00923515	A
OR4N4	0.11218848	0.00974244	A
LCE2A	0.11077427	0.01070904	A
SLC15A1	0.10536298	0.01523816	A
TNNT3	0.10482582	0.01576858	A
OR4L1	0.10310658	0.01757661	A
PRSS2	0.10282783	0.01788622	A
ZACN	0.10275602	0.01796675	A
CLDN22	0.10136672	0.01958828	A
KLK9	0.08771374	0.04354485	A
MAGEC3	0.08459538	0.05160385	A
OR5I1	0.08293769	0.05637193	A

RBMY3AP	0.08275392	0.05692232	A
TTTY23	0.08105608	0.06222143	A
OR4C15	0.08054143	0.06390585	A
ABHD16B	0.08037348	0.0644636	A
KRTAP19-6	0.07945677	0.06757897	A
NPFF	0.0788171	0.06982519	A
KRTAP9-8	0.0765829	0.07815457	A
OR7G3	0.07649797	0.07848646	A
MYH16	0.07621894	0.07958489	A
OR10A6	0.0760238	0.08036051	A
CALML6	0.07372102	0.08998329	A
HULC	0.07160311	0.09963011	A
OR5M10	0.07122019	0.10145906	A
PRAMEF5	0.06712129	0.12274814	A
IRX6	0.06457618	0.13762457	A
KRTAP4-8	0.06446496	0.13830497	A
OR8I2	0.06218164	0.15285123	A
OR5D18	0.06181707	0.15527749	A
OR5D14	0.06163507	0.15649955	A
KRTAP9-9	0.06118793	0.15953288	A
OR10X1	0.0604755	0.16445719	A
C11orf86	0.06045169	0.16462376	A
TTTY6	0.06005401	0.1674238	A
PTTG2	0.05717948	0.18873254	A
SFT2D3	0.05714982	0.18896233	A
OR5T1	0.05637164	0.1950654	A
CDRT7	0.05637002	0.19507827	A
APOB	0.05516505	0.20481155	A
OR4C13	0.05454005	0.20999657	A
OR8K3	0.05349384	0.21888683	A
MEG8	0.05210751	0.23107809	A
HIST2H3D	0.05181862	0.23367798	A
OR52R1	0.05145235	0.23700392	A
OR5R1	0.04969969	0.25338034	A
OR51A2	0.04943243	0.25594499	A
CSPG4P1Y	0.04935754	0.2566669	A
DEFB124	0.04836355	0.26638147	A
OR8K1	0.04783479	0.27165087	A
KRTAP9-4	0.04634125	0.28691714	A
OR10AG1	0.04485471	0.30267512	A
FADS6	0.04426227	0.30911221	A
LCE3E	0.03857893	0.37540924	A
OR4K5	0.03751509	0.38872798	A
SPHAR	0.03726569	0.39189129	A
TTTY5	0.0369491	0.39592932	A
HIST1H2BF	0.03650267	0.4016658	A

OR5H6	0.03626336	0.40476118	A
KRTAP9-7	0.03582951	0.41040903	A
OR5T3	0.03581794	0.41056026	A
OR8K5	0.03346334	0.44202371	A
DEFB104B	0.03250173	0.45525874	A
CXorf51A	0.0316226	0.46755081	A
OR4X1	0.02997027	0.49114233	A
DEFB107A	0.0298807	0.49243915	A
CPN1	0.02887683	0.50709799	A
DPH3P1	0.02717874	0.53240461	A
FTHL17	0.02446616	0.57411092	A
OR5D13	0.02422288	0.57792547	A
OR8H1	0.02364532	0.58702875	A
TTTY8	0.02034853	0.64021323	A
TTTY1	0.0195968	0.65261647	A
KRTAP19-2	0.01949236	0.65434747	A
KRTAP19-7	0.01888085	0.66451927	A
KRTAP6-2	0.01832997	0.67373564	A
PRAMEF4	0.01785089	0.6817907	A
DUX4L4	0.01590159	0.71493162	A
OR10R2	0.01496222	0.73110036	A
OR5M3	0.01472198	0.73525496	A
OR2L8	0.01320773	0.76161552	A
SLC30A2	0.01117828	0.79737654	A
VSIG2	0.00975839	0.82265623	A
OR9G4	0.00467366	0.91451749	A
OR4C16	0.00441147	0.91929607	A
OR6C65	0.00364002	0.93337284	A
RBMY1B	-0.0002702	0.99504909	B
SNORA78	-0.0005095	0.99066436	B
ANKRD36BF	-0.0009008	0.98349345	B
OR9G1	-0.0023658	0.95666796	B
KRTAP22-2	-0.0083172	0.84850419	B
TSSK2	-0.0148366	0.73327252	B
RPL23AP32	-0.0158949	0.7150465	B
LCE1D	-0.0202733	0.64145049	B
OR4C1P	-0.0244246	0.57476116	B
OR8H3	-0.0255969	0.55653902	B
OR5AR1	-0.0382301	0.37974564	B
GAB4	-0.0407652	0.34893388	B
SCARNA17	-0.0414546	0.34083747	B
KRTAP19-4	-0.0414546	0.34083747	B
HIST1H2AK	-0.0466755	0.28345166	B
SLC38A4	-0.0518751	0.23316765	B
HYMAI	-0.0580984	0.18171432	B
OR4S2	-0.0690815	0.11216916	B

UGT1A8	-0.0694385	0.11032186	B
DND1	-0.0804548	0.06419321	B
RPS15AP10	-0.0805765	0.06378974	B
OR7E12P	-0.0823105	0.0582688	B
PIPOX	-0.0866177	0.04624737	B
PRLR	-0.0873583	0.04440623	B
OR3A4P	-0.0882298	0.04231907	B
PIGR	-0.0895036	0.03941782	B
MTMR9LP	-0.0903893	0.03750132	B
PPP1R14D	-0.094259	0.03002851	B
OR8H2	-0.1004867	0.02068013	B
C2orf54	-0.1012978	0.01967189	B
CYP8B1	-0.1014243	0.01951859	B
SNORA59B	-0.1057921	0.01482575	B
NPHS1	-0.1063925	0.01426519	B
BPY2B	-0.1185803	0.00627362	B
GLOD5	-0.1226142	0.00470157	B
MAOA	-0.1243823	0.00413235	B
REG1A	-0.1263477	0.00357335	B
FMO6P	-0.1347955	0.00187021	B
LRRC71	-0.1363428	0.00165441	B
CWH43	-0.1466692	0.00070677	B
IZUMO1	-0.1516763	0.0004585	B
RNF138P1	-0.1524109	0.00042981	B
HTATSF1P2	-0.1552513	0.00033388	B
TSPAN1	-0.1608119	0.00020109	B
GC	-0.1621418	0.00017768	B
SVOP	-0.1623776	0.00017381	B
LECT2	-0.1689615	9.28E-05	B
C2orf40	-0.1728808	6.31E-05	B
MPV17L	-0.1757871	4.72E-05	B
B3GNT3	-0.1812397	2.70E-05	B
BHLHA15	-0.1836662	2.09E-05	B
CYP17A1	-0.1890813	1.17E-05	B
MUM1L1	-0.190195	1.04E-05	B
SRRM4	-0.1927247	7.88E-06	B
AZGP1P1	-0.1964809	5.19E-06	B
GCNT3	-0.1973475	4.70E-06	B
RFPL4A	-0.1982017	4.27E-06	B
TRPC7	-0.2018034	2.82E-06	B
GALNT9	-0.2019601	2.77E-06	B
RBP4	-0.2055548	1.82E-06	B
MLIP	-0.2068038	1.57E-06	B
BMP5	-0.206954	1.55E-06	B
OGDHL	-0.2090239	1.21E-06	B
C10orf67	-0.2119088	8.52E-07	B

HES5	-0.2162035	5.03E-07	B
UNC5D	-0.2163042	4.97E-07	B
RGS7BP	-0.2185416	3.76E-07	B
MYH8	-0.2190107	3.54E-07	B
FLJ22763	-0.2209621	2.77E-07	B
OPCML	-0.2225216	2.27E-07	B
C2orf72	-0.2225953	2.25E-07	B
AKR1C4	-0.2234062	2.03E-07	B
IFNA22P	-0.2238581	1.91E-07	B
ARSE	-0.2264148	1.37E-07	B
VIP	-0.229944	8.65E-08	B
TEX15	-0.2312415	7.28E-08	B
RMST	-0.2327403	5.96E-08	B
ACOT6	-0.2349403	4.43E-08	B
SLC7A13	-0.2355062	4.10E-08	B
CCL16	-0.2364705	3.60E-08	B
PTGER3	-0.2388015	2.62E-08	B
SLC23A1	-0.2414281	1.82E-08	B
MGST1	-0.2428712	1.49E-08	B
OVCH1	-0.2429545	1.47E-08	B
LPPR5	-0.2431505	1.43E-08	B
UGT2B10	-0.2431904	1.42E-08	B
CDH9	-0.2435967	1.34E-08	B
SST	-0.2436089	1.34E-08	B
OR2G6	-0.244369	1.20E-08	B
PSORS1C3	-0.2465879	8.79E-09	B
HMGCLL1	-0.2485635	6.63E-09	B
TRIM71	-0.249261	5.99E-09	B
ESRRG	-0.249664	5.66E-09	B
OR2T2	-0.2509201	4.71E-09	B
PNPLA3	-0.2509704	4.68E-09	B
FABP1	-0.2520748	3.99E-09	B
GGTLC2	-0.2524475	3.77E-09	B
CAPN14	-0.2588055	1.47E-09	B
KCNK3	-0.2595373	1.32E-09	B
SERPINF2	-0.2616242	9.62E-10	B
GBP7	-0.2641041	6.58E-10	B
ANGPTL3	-0.2656066	5.22E-10	B
COX6A2	-0.2663594	4.65E-10	B
FBXL21	-0.2664974	4.55E-10	B
ZNF804B	-0.2692501	2.96E-10	B
STH	-0.2712781	2.15E-10	B
CNNM1	-0.2718075	1.98E-10	B
MAEL	-0.2719723	1.93E-10	B
F11	-0.2744852	1.29E-10	B
UPP2	-0.2753633	1.12E-10	B

C6	-0.2758862	1.03E-10	B
SLC2A5	-0.2765466	9.28E-11	B
KCNV1	-0.2777751	7.60E-11	B
OR14I1	-0.2792801	5.95E-11	B
CACNA1E	-0.2812353	4.32E-11	B
PPARGC1A	-0.281274	4.29E-11	B
PROC	-0.283663	2.89E-11	B
IL22RA1	-0.2850085	2.31E-11	B
GGT3P	-0.286893	1.68E-11	B
HIST1H2AA	-0.2874321	1.54E-11	B
OVOL1	-0.2901888	9.63E-12	B
IL1RAPL2	-0.2915975	7.56E-12	B
KCNN1	-0.2993509	1.96E-12	B
MYOC	-0.2998111	1.81E-12	B
ALPI	-0.3038803	8.74E-13	B
HIST1H2APS	-0.3048713	7.31E-13	B
C1orf95	-0.3059402	6.02E-13	B
GRM3	-0.3062757	5.67E-13	B
ATP13A4	-0.306592	5.35E-13	B
MARVELD3	-0.3071329	4.85E-13	B
C21orf62	-0.3092205	3.31E-13	B
IHH	-0.3101377	2.80E-13	B
AGT	-0.3111654	2.31E-13	B
PCGEM1	-0.3113993	2.21E-13	B
SLC5A9	-0.3116107	2.13E-13	B
NECAB2	-0.3118582	2.03E-13	B
FAM65C	-0.3123191	1.87E-13	B
GRIA4	-0.3148659	1.16E-13	B
AZGP1	-0.3151413	1.10E-13	B
CCDC146	-0.3160225	9.34E-14	B
NGEF	-0.316609	8.36E-14	B
CYP4Z2P	-0.316838	8.00E-14	B
F2RL3	-0.3184798	5.86E-14	B
CECR3	-0.3195981	4.74E-14	B
ZNF385B	-0.3198993	4.47E-14	B
MLNR	-0.3214985	3.29E-14	B
CASQ2	-0.3222544	2.85E-14	B
ARPP21	-0.3223083	2.82E-14	B
CHRM3	-0.3236497	2.17E-14	B
LINC00173	-0.3241785	1.96E-14	B
FAM81B	-0.3248578	1.72E-14	B
CYP3A4	-0.3249944	1.67E-14	B
AMACR	-0.327166	1.09E-14	B
SLC9A3	-0.3278369	9.59E-15	B
SLC47A2	-0.3279011	9.47E-15	B
RERGL	-0.3295752	6.80E-15	B

PRAP1	-0.3297227	6.61E-15	B
ZNF366	-0.3303157	5.87E-15	B
SSTR1	-0.3313581	4.77E-15	B
ANO4	-0.3316736	4.48E-15	B
EPHA7	-0.3318462	4.33E-15	B
ABCC2	-0.3323677	3.90E-15	B
RNF186	-0.3325412	3.77E-15	B
ASXL3	-0.3339516	2.84E-15	B
SHISA9	-0.3340976	2.76E-15	B
LINC00113	-0.3343465	2.62E-15	B
FABP4	-0.3345758	2.50E-15	B
OR2T5	-0.3351106	2.25E-15	B
FOLR1	-0.3361035	1.84E-15	B
LGSN	-0.3361964	1.80E-15	B
EDN2	-0.3365936	1.66E-15	B
CRYAB	-0.3394271	9.32E-16	B
SEMA5B	-0.3396258	8.94E-16	B
NR1H4	-0.3397564	8.71E-16	B
TEX11	-0.3402479	7.87E-16	B
IYD	-0.3417384	5.78E-16	B
MYOT	-0.3418572	5.64E-16	B
GPX3	-0.342269	5.18E-16	B
LIPC	-0.3442664	3.42E-16	B
CHST9	-0.3447315	3.10E-16	B
G6PC2	-0.3455389	2.62E-16	B
SPON1	-0.3461693	2.29E-16	B
PLA2G12B	-0.3474783	1.74E-16	B
CIB4	-0.347953	1.57E-16	B
SLITRK5	-0.3506158	8.93E-17	B
MORN5	-0.3512138	7.86E-17	B
AQP4	-0.3513476	7.63E-17	B
MOGAT3	-0.3514663	7.44E-17	B
AOX1	-0.3525111	5.95E-17	B
RGN	-0.3525367	5.91E-17	B
RAB19	-0.352888	5.48E-17	B
CDH16	-0.3535862	4.71E-17	B
NLRP11	-0.3545071	3.86E-17	B
CRIP3	-0.3552625	3.28E-17	B
UGT3A1	-0.3557083	2.98E-17	B
HGD	-0.3564464	2.53E-17	B
FAM151A	-0.3565862	2.46E-17	B
SLC5A11	-0.3571205	2.19E-17	B
C4orf19	-0.3595208	1.29E-17	B
FXYD2	-0.3599165	1.18E-17	B
OR10Q1	-0.3605899	1.02E-17	B
SOX6	-0.3639376	4.83E-18	B

F2RL1	-0.3640445	4.71E-18	B
EDN1	-0.3641116	4.64E-18	B
ASPDH	-0.3658082	3.17E-18	B
AGXT	-0.3663757	2.79E-18	B
DNASE1L3	-0.3667048	2.59E-18	B
GGTLC1	-0.3667361	2.57E-18	B
PALM3	-0.3677057	2.06E-18	B
FABP3	-0.3686933	1.65E-18	B
ENPP7	-0.3693063	1.43E-18	B
ABCC6P1	-0.3704665	1.10E-18	B
OIT3	-0.3704872	1.09E-18	B
IP6K3	-0.3721616	7.44E-19	B
PLG	-0.3725904	6.74E-19	B
GSTA7P	-0.3726444	6.65E-19	B
CNDP1	-0.3737845	5.11E-19	B
C6orf223	-0.3760688	3.00E-19	B
KLF15	-0.3771464	2.33E-19	B
SEC14L6	-0.3772769	2.26E-19	B
CYP3A7	-0.3776143	2.09E-19	B
UGT1A6	-0.3777699	2.01E-19	B
CCDC85A	-0.3780105	1.90E-19	B
UGT2B11	-0.3786355	1.64E-19	B
HOGA1	-0.3808827	9.65E-20	B
ANPEP	-0.3818677	7.63E-20	B
CHST13	-0.3820998	7.22E-20	B
AMIGO1	-0.3849368	3.65E-20	B
ADSSL1	-0.3849745	3.62E-20	B
ANO3	-0.3854062	3.26E-20	B
LRRC66	-0.3859757	2.84E-20	B
KCNJ16	-0.3871769	2.12E-20	B
USP2	-0.3878665	1.80E-20	B
DGCR5	-0.3878873	1.79E-20	B
SERPINA7	-0.3886782	1.47E-20	B
AKR1C1	-0.3888072	1.43E-20	B
CD300LG	-0.3891382	1.32E-20	B
HRASLS2	-0.3907292	8.91E-21	B
FGFBP2	-0.3908525	8.65E-21	B
A4GNT	-0.3930115	5.07E-21	B
PAH	-0.3931257	4.93E-21	B
GRIK3	-0.3934028	4.60E-21	B
MASP1	-0.3936944	4.28E-21	B
CGREF1	-0.3937996	4.17E-21	B
ILDR2	-0.3943749	3.62E-21	B
MOGAT1	-0.3955024	2.73E-21	B
RIMKLA	-0.3955327	2.71E-21	B
GDA	-0.3981884	1.39E-21	B

ALDH4A1	-0.3990373	1.12E-21	B
SLC34A1	-0.4004409	7.86E-22	B
FAM150B	-0.4010965	6.65E-22	B
METTL7B	-0.4011898	6.49E-22	B
GUCA2B	-0.4012388	6.41E-22	B
ABO	-0.4015595	5.91E-22	B
ETNK2	-0.4035473	3.55E-22	B
KCTD16	-0.4040908	3.09E-22	B
IQSEC3	-0.4047321	2.62E-22	B
TMEM132D	-0.4049157	2.50E-22	B
SORBS2	-0.4057765	2.00E-22	B
SCN4B	-0.4059182	1.93E-22	B
ABCB1	-0.4063725	1.71E-22	B
AFM	-0.4066065	1.61E-22	B
CDCA2	-0.4070377	1.44E-22	B
KCNH6	-0.408177	1.07E-22	B
TM6SF2	-0.4083471	1.02E-22	B
ZCCHC16	-0.4086583	9.44E-23	B
CDHR2	-0.4086894	9.37E-23	B
AIF1L	-0.40876	9.20E-23	B
PDZK1IP1	-0.4092273	8.14E-23	B
TTC22	-0.4103849	6.01E-23	B
GAMT	-0.4104292	5.94E-23	B
GABRG1	-0.4105549	5.74E-23	B
MFAP3L	-0.4108946	5.25E-23	B
PDK4	-0.4112126	4.83E-23	B
AJAP1	-0.4114167	4.58E-23	B
SLCO2A1	-0.4121012	3.82E-23	B
DPP4	-0.4122741	3.65E-23	B
HMGCS2	-0.412806	3.17E-23	B
ASPG	-0.413817	2.42E-23	B
ZYG11A	-0.4151955	1.68E-23	B
PTPRB	-0.4153008	1.63E-23	B
GAL3ST1	-0.41563	1.49E-23	B
GSTA2	-0.4163897	1.22E-23	B
GPD1	-0.4168888	1.07E-23	B
TMEM200A	-0.4170731	1.01E-23	B
NLGN1	-0.4183697	7.15E-24	B
ENPP5	-0.4186978	6.54E-24	B
UBD	-0.4194516	5.34E-24	B
SLC22A8	-0.4200069	4.59E-24	B
HAVCR1	-0.4201591	4.40E-24	B
TCL6	-0.4207999	3.70E-24	B
MME	-0.4209084	3.59E-24	B
C1QL4	-0.4209688	3.53E-24	B
ENPP2	-0.4213726	3.16E-24	B

SLC13A2	-0.4223298	2.44E-24	B
NRTN	-0.4227932	2.15E-24	B
FLT1	-0.4236055	1.72E-24	B
SLC22A7	-0.4237859	1.63E-24	B
CREB3L3	-0.4246106	1.30E-24	B
CES2	-0.4263232	8.11E-25	B
COL19A1	-0.4277052	5.52E-25	B
DSCAML1	-0.4277898	5.39E-25	B
APOLD1	-0.4278467	5.31E-25	B
MYO7B	-0.4279169	5.20E-25	B
HNF4G	-0.4284608	4.47E-25	B
SLC6A12	-0.4302112	2.74E-25	B
LIN7A	-0.4302705	2.69E-25	B
PGA3	-0.4308968	2.26E-25	B
NAPSA	-0.4314261	1.94E-25	B
DEPDC7	-0.4315155	1.90E-25	B
ALDH8A1	-0.4319882	1.66E-25	B
CASP12	-0.4326907	1.36E-25	B
SLC4A4	-0.4334923	1.08E-25	B
SALL1	-0.4347842	7.50E-26	B
MYO3A	-0.4362229	4.97E-26	B
DPF3	-0.4368094	4.20E-26	B
TRIM55	-0.4378098	3.15E-26	B
TRIM6	-0.43802	2.96E-26	B
IL17RB	-0.4387303	2.41E-26	B
C14orf180	-0.4391222	2.16E-26	B
OR2T10	-0.4391615	2.13E-26	B
FAM196B	-0.4398977	1.72E-26	B
COL23A1	-0.4416625	1.03E-26	B
PCA3	-0.4422294	8.73E-27	B
FLRT3	-0.4428802	7.21E-27	B
PRODH	-0.4429904	6.98E-27	B
FAM107A	-0.4434808	6.05E-27	B
CCDC64	-0.444189	4.91E-27	B
ALMS1P	-0.4442932	4.76E-27	B
CTXN3	-0.4443874	4.63E-27	B
GABRB3	-0.4444438	4.56E-27	B
FGFR3	-0.4448797	4.01E-27	B
KDR	-0.4450032	3.86E-27	B
FAM110C	-0.4450389	3.82E-27	B
COBL	-0.4455938	3.24E-27	B
SLC2A9	-0.4459099	2.95E-27	B
HNF4A	-0.4465723	2.43E-27	B
MLXIPL	-0.4476954	1.74E-27	B
KLHDC7A	-0.4478748	1.65E-27	B
ABAT	-0.448704	1.29E-27	B

KLKB1	-0.449261	1.09E-27	B
TMEM176A	-0.449483	1.02E-27	B
PAX2	-0.4495904	9.87E-28	B
KBTBD11	-0.4498602	9.10E-28	B
TMEM176B	-0.4503579	7.84E-28	B
EVPLL	-0.4511501	6.18E-28	B
MPP6	-0.4511577	6.16E-28	B
AACSP1	-0.4512417	6.01E-28	B
FMO2	-0.4516582	5.30E-28	B
HPN	-0.4528657	3.68E-28	B
RBP7	-0.4533057	3.22E-28	B
PAQR5	-0.4533473	3.18E-28	B
STOML3	-0.4540143	2.60E-28	B
GYPA	-0.4544883	2.25E-28	B
GHR	-0.4554895	1.66E-28	B
CXCL14	-0.4555539	1.62E-28	B
EDAR	-0.4564299	1.24E-28	B
GLTPD2	-0.4564318	1.24E-28	B
CD36	-0.4566892	1.15E-28	B
ARHGEF37	-0.4589224	5.76E-29	B
COL25A1	-0.4590913	5.47E-29	B
DPY19L2P2	-0.4598451	4.33E-29	B
EXOC3L4	-0.4598876	4.27E-29	B
ENTPD5	-0.4602463	3.82E-29	B
CLRN3	-0.4606292	3.40E-29	B
TNFAIP6	-0.4607548	3.27E-29	B
REN	-0.4613603	2.70E-29	B
PNMA2	-0.4614544	2.63E-29	B
NEBL	-0.4621076	2.14E-29	B
GDF6	-0.4625382	1.87E-29	B
TREH	-0.4626001	1.84E-29	B
ACADM	-0.462608	1.83E-29	B
IL17RD	-0.4627963	1.73E-29	B
SLC34A3	-0.4633166	1.47E-29	B
KIF12	-0.463984	1.19E-29	B
TCN2	-0.4640832	1.15E-29	B
MAOB	-0.464119	1.14E-29	B
UCN3	-0.4642311	1.10E-29	B
HRH2	-0.4652417	8.01E-30	B
CRB3	-0.4662196	5.88E-30	B
TLR3	-0.4662314	5.86E-30	B
PTH1R	-0.4664514	5.46E-30	B
BARX2	-0.466753	4.96E-30	B
ALPK2	-0.4669135	4.72E-30	B
ENPP3	-0.4673226	4.14E-30	B
ALDH6A1	-0.4674134	4.02E-30	B

ABCC6	-0.4674996	3.92E-30	B
ACOT4	-0.4689097	2.50E-30	B
ENTPD8	-0.4691748	2.30E-30	B
SERPINA6	-0.4691763	2.29E-30	B
C14orf105	-0.4698456	1.85E-30	B
PDZD3	-0.4707256	1.40E-30	B
ANXA13	-0.4709013	1.32E-30	B
ELOVL7	-0.4709307	1.31E-30	B
COL18A1-A	-0.471303	1.16E-30	B
BHMT2	-0.471685	1.03E-30	B
DOC2A	-0.4725539	7.74E-31	B
TMEM139	-0.4725958	7.64E-31	B
EDNRB	-0.4726913	7.41E-31	B
ADH6	-0.4727647	7.23E-31	B
DPYS	-0.4730216	6.66E-31	B
NPY6R	-0.4735625	5.59E-31	B
FUT3	-0.4740885	4.71E-31	B
ABCG2	-0.4741991	4.54E-31	B
FCAMR	-0.4743833	4.28E-31	B
OR7E47P	-0.4755101	2.96E-31	B
AGMO	-0.4763236	2.27E-31	B
DAO	-0.4764793	2.16E-31	B
PNMA6A	-0.4768393	1.92E-31	B
FRMD1	-0.4769112	1.87E-31	B
AKR7L	-0.476932	1.86E-31	B
EGOT	-0.4775025	1.54E-31	B
CCL15	-0.4777969	1.40E-31	B
GLIS1	-0.4781523	1.25E-31	B
GYLTL1B	-0.4797844	7.27E-32	B
C2orf73	-0.4804229	5.88E-32	B
ENAM	-0.4806552	5.45E-32	B
SCGB1D2	-0.4812175	4.52E-32	B
SLC16A4	-0.4814419	4.19E-32	B
SULT1C4	-0.4815402	4.06E-32	B
AR	-0.4821991	3.26E-32	B
SEMA3D	-0.4826167	2.83E-32	B
PRODH2	-0.4845297	1.49E-32	B
ALDOB	-0.4870383	6.39E-33	B
FZD5	-0.487066	6.33E-33	B
ACY3	-0.4872913	5.87E-33	B
RBP5	-0.4875809	5.32E-33	B
SLITRK4	-0.4880619	4.52E-33	B
CA4	-0.4889815	3.30E-33	B
EMCN	-0.4897082	2.57E-33	B
ACSM5	-0.4898929	2.42E-33	B
SLC17A2	-0.4913485	1.47E-33	B

ADCY5	-0.4915645	1.36E-33	B
CYS1	-0.4918044	1.25E-33	B
FRAS1	-0.4932207	7.69E-34	B
CDHR5	-0.4941944	5.48E-34	B
TM4SF5	-0.4947843	4.47E-34	B
UGT2B7	-0.4948411	4.38E-34	B
ZNF704	-0.4950931	4.01E-34	B
IQUB	-0.4953714	3.64E-34	B
SYT9	-0.4955373	3.44E-34	B
SDPR	-0.4956918	3.26E-34	B
ALPL	-0.4957548	3.19E-34	B
GGT8P	-0.4963388	2.60E-34	B
TMEM37	-0.496648	2.33E-34	B
ZSWIM5	-0.497052	2.02E-34	B
CHDH	-0.4973896	1.80E-34	B
CIT	-0.4980171	1.44E-34	B
PLS1	-0.4986366	1.16E-34	B
ATOH8	-0.4989456	1.04E-34	B
FTCD	-0.4998718	7.50E-35	B
ATP11A	-0.5001037	6.91E-35	B
VCAM1	-0.500494	6.01E-35	B
RASSF6	-0.5015123	4.19E-35	B
TMEM150C	-0.5020287	3.49E-35	B
KCNJ3	-0.502344	3.11E-35	B
ESPN	-0.5025459	2.90E-35	B
TRHDE	-0.5038108	1.84E-35	B
ENPEP	-0.5038581	1.81E-35	B
DHDH	-0.504641	1.37E-35	B
LIFR	-0.5049164	1.24E-35	B
ZNF711	-0.5070768	5.68E-36	B
ERBB3	-0.5074595	4.95E-36	B
TSPAN12	-0.5076795	4.57E-36	B
SLC6A3	-0.5078359	4.31E-36	B
PPAP2B	-0.5079214	4.18E-36	B
WDR72	-0.5082992	3.65E-36	B
CES3	-0.5096841	2.20E-36	B
TMEM72	-0.5097812	2.12E-36	B
CASC1	-0.5109452	1.39E-36	B
QRFPR	-0.5112101	1.26E-36	B
BAIAP2L2	-0.5114883	1.14E-36	B
HSD3B2	-0.5124756	7.89E-37	B
TMED6	-0.5137237	4.98E-37	B
CYB5A	-0.5155769	2.50E-37	B
SAMD5	-0.5156073	2.47E-37	B
PCK1	-0.5159483	2.18E-37	B
USH1C	-0.5163146	1.90E-37	B

SYDE2	-0.5167127	1.64E-37	B
XYLB	-0.5177758	1.10E-37	B
TRPV4	-0.5179844	1.02E-37	B
CABP1	-0.5191486	6.55E-38	B
HHLA2	-0.5193453	6.08E-38	B
TFEC	-0.5199417	4.86E-38	B
DNAJC22	-0.5201326	4.52E-38	B
CLDN10	-0.5202244	4.36E-38	B
GJB1	-0.520619	3.76E-38	B
FBXL16	-0.5210753	3.16E-38	B
UGT2A3	-0.5225559	1.80E-38	B
TLN2	-0.5237479	1.14E-38	B
KCNK5	-0.5241581	9.75E-39	B
HLF	-0.5248954	7.34E-39	B
VIL1	-0.5251061	6.77E-39	B
AGTR1	-0.5254824	5.86E-39	B
HNF1A-AS1	-0.5257966	5.19E-39	B
RAB17	-0.5266932	3.67E-39	B
PDGFD	-0.5267727	3.56E-39	B
SCN4A	-0.5288343	1.60E-39	B
CYP2J2	-0.5289449	1.53E-39	B
GGT1	-0.5291435	1.42E-39	B
EMX2	-0.5308362	7.32E-40	B
ACMSD	-0.531064	6.69E-40	B
TLL1	-0.5311327	6.51E-40	B
BHMT	-0.5324834	3.83E-40	B
NYX	-0.5338596	2.22E-40	B
AQP7	-0.5343658	1.82E-40	B
SLC37A4	-0.5357271	1.06E-40	B
TMEM125	-0.5359916	9.52E-41	B
PAIP2B	-0.5366396	7.35E-41	B
SLC28A1	-0.5372258	5.81E-41	B
MYOM3	-0.5377208	4.77E-41	B
RGS9	-0.5385545	3.41E-41	B
CCDC150	-0.5410025	1.27E-41	B
CRYZ	-0.5414737	1.05E-41	B
LHFPL3	-0.5428642	5.95E-42	B
ASB17	-0.5434825	4.62E-42	B
DMGDH	-0.5446387	2.88E-42	B
ALDH1L1	-0.5452561	2.23E-42	B
NTN4	-0.545836	1.76E-42	B
AFF3	-0.5461903	1.52E-42	B
BTNL9	-0.5473643	9.34E-43	B
DIRAS2	-0.5476181	8.41E-43	B
GPT	-0.5477553	7.94E-43	B
ANK3	-0.547905	7.46E-43	B

PLIN2	-0.5484888	5.86E-43	B
HNF1A	-0.5485926	5.61E-43	B
AKR7A3	-0.5508176	2.21E-43	B
CLEC18C	-0.5526216	1.04E-43	B
EMX2OS	-0.5530251	8.74E-44	B
CLDN2	-0.5534679	7.25E-44	B
SLC2A2	-0.5535649	6.96E-44	B
PRUNE2	-0.5544189	4.85E-44	B
TRIM15	-0.5554345	3.15E-44	B
LRRC19	-0.5565527	1.95E-44	B
PANK1	-0.5578637	1.11E-44	B
DHTKD1	-0.5579932	1.05E-44	B
ADM2	-0.5581813	9.71E-45	B
CLVS2	-0.5584676	8.59E-45	B
NRXN2	-0.5591887	6.30E-45	B
KLHL32	-0.5606531	3.34E-45	B
KL	-0.5610949	2.76E-45	B
RUNDC3B	-0.5627651	1.33E-45	B
ECHDC3	-0.5630384	1.18E-45	B
CRYM	-0.563275	1.07E-45	B
LRRK2	-0.563577	9.35E-46	B
FREM2	-0.5649524	5.11E-46	B
NXNL2	-0.5652912	4.40E-46	B
ARSF	-0.5653383	4.31E-46	B
KHK	-0.5661951	2.96E-46	B
DAB2	-0.5669493	2.12E-46	B
CLEC18B	-0.5678568	1.42E-46	B
SLC39A5	-0.5685011	1.06E-46	B
SLCO4C1	-0.5694939	6.83E-47	B
NOSTRIN	-0.5704864	4.38E-47	B
SPATA18	-0.5708931	3.65E-47	B
FAAH	-0.5711398	3.27E-47	B
ABHD6	-0.5713146	3.02E-47	B
GLYAT	-0.5741403	8.43E-48	B
TMEM82	-0.5758619	3.85E-48	B
MYL3	-0.5766667	2.66E-48	B
DMRTA1	-0.5767934	2.51E-48	B
IMPA2	-0.576956	2.33E-48	B
C1orf115	-0.5771198	2.16E-48	B
SHMT1	-0.5783943	1.20E-48	B
CRYL1	-0.5786692	1.06E-48	B
SLC17A4	-0.578717	1.04E-48	B
AMN	-0.5799019	6.00E-49	B
MGAM	-0.5801771	5.28E-49	B
EPHX2	-0.5825835	1.72E-49	B
KCNJ15	-0.582718	1.62E-49	B

G6PC	-0.5836189	1.06E-49	B
HRSP12	-0.5838386	9.58E-50	B
ANKS4B	-0.5846061	6.68E-50	B
PTH2R	-0.5851426	5.19E-50	B
SLC1A1	-0.5856495	4.08E-50	B
TMIGD1	-0.5860438	3.39E-50	B
SLC16A12	-0.5866078	2.59E-50	B
SLC26A9	-0.5870501	2.10E-50	B
PBLD	-0.5890351	8.17E-51	B
SLC6A18	-0.592816	1.33E-51	B
PKLR	-0.5939664	7.59E-52	B
SLC22A4	-0.5940009	7.46E-52	B
PXMP2	-0.5941206	7.04E-52	B
AQP1	-0.5943982	6.15E-52	B
UPB1	-0.5949032	4.81E-52	B
ASRGL1	-0.5955255	3.55E-52	B
LYG1	-0.5983748	8.75E-53	B
SLC3A1	-0.5985071	8.19E-53	B
AGXT2	-0.5987086	7.42E-53	B
STK32B	-0.6010671	2.30E-53	B
C11orf54	-0.601498	1.86E-53	B
SLC7A9	-0.6017439	1.64E-53	B
SLC23A3	-0.6019805	1.46E-53	B
TINAG	-0.6025655	1.09E-53	B
TMEM38B	-0.6035277	6.71E-54	B
ACSM2B	-0.6045789	3.95E-54	B
TMEM171	-0.6065855	1.43E-54	B
FRMD3	-0.6073154	9.86E-55	B
NLRP6	-0.6094109	3.38E-55	B
AVPR1B	-0.6108511	1.61E-55	B
TRIM10	-0.6124421	7.06E-56	B
FMO1	-0.6126376	6.38E-56	B
CMBL	-0.6134403	4.20E-56	B
EHHADH	-0.6156224	1.34E-56	B
A1CF	-0.6156374	1.33E-56	B
LRP2	-0.6160283	1.08E-56	B
FBP1	-0.6162622	9.58E-57	B
SLC22A13	-0.6168091	7.19E-57	B
AMDHD1	-0.6177975	4.26E-57	B
CUBN	-0.6180327	3.76E-57	B
C1orf210	-0.6189408	2.33E-57	B
PKHD1	-0.6205082	1.01E-57	B
SLC6A19	-0.6221839	4.11E-58	B
SLC5A8	-0.622492	3.48E-58	B
FUT6	-0.6232892	2.27E-58	B
ACAD11	-0.6243807	1.26E-58	B

ACSM2A	-0.6259663	5.30E-59	B
SLC22A2	-0.6260534	5.06E-59	B
SLC5A12	-0.6282373	1.53E-59	B
MAP7	-0.6296361	7.08E-60	B
CLCN5	-0.6301653	5.28E-60	B
ACADL	-0.6305005	4.39E-60	B
LGALS2	-0.6313882	2.68E-60	B
ASPA	-0.6318757	2.04E-60	B
MAPT	-0.6320839	1.82E-60	B
APOM	-0.634015	6.18E-61	B
SLC10A2	-0.6363867	1.62E-61	B
C9orf66	-0.638044	6.33E-62	B
DDAH1	-0.6390145	3.64E-62	B
TAL2	-0.6423905	5.21E-63	B
PDZK1	-0.6427757	4.17E-63	B
SLC6A13	-0.6443378	1.68E-63	B
BBOX1	-0.64911	1.01E-64	B
SLC17A3	-0.649112	1.01E-64	B
SLC17A1	-0.6499819	6.04E-65	B
GATM	-0.6505441	4.32E-65	B
DDC	-0.6520024	1.80E-65	B
GLYATL1	-0.653053	9.58E-66	B
SLC13A1	-0.6548164	3.30E-66	B
PHYHIPL	-0.6551392	2.71E-66	B
SMTNL2	-0.6567091	1.04E-66	B
MSRA	-0.6638261	1.27E-68	B
SLC5A1	-0.6659375	3.34E-69	B
SLC16A9	-0.6680981	8.47E-70	B
AGMAT	-0.6684148	6.91E-70	B
ACE2	-0.6692654	4.01E-70	B
CYP4A22	-0.6715482	9.21E-71	B
SLC22A11	-0.6736525	2.35E-71	B
ACAA2	-0.6744333	1.41E-71	B
NAT8	-0.6752837	8.06E-72	B
SLC25A48	-0.6755743	6.66E-72	B
SCGN	-0.6770561	2.51E-72	B
GBA3	-0.6802087	3.07E-73	B
NPR3	-0.6830994	4.38E-74	B
SLC22A6	-0.6832625	3.92E-74	B
SLC47A1	-0.6879475	1.59E-75	B
TMEM174	-0.6923443	7.40E-77	B
SLC5A10	-0.6969226	2.87E-78	B
HAO2	-0.697599	1.76E-78	B
GIPC2	-0.6978731	1.45E-78	B
TRPM3	-0.7003179	2.47E-79	B
SLC22A12	-0.7027565	4.16E-80	B

MIOX	-0.706001	3.77E-81	B
CYP4A11	-0.7075707	1.17E-81	B
TMEM27	-0.7117751	4.86E-83	B
SLC27A2	-0.7143898	6.53E-84	B
NAT8B	-0.7212749	2.97E-86	B
SLC22A24	-0.7231611	6.58E-87	B
