# Supplementary material for: Comprehensive Characterization of Metabolism-Associated Subtypes of Renal Cell Carcinoma to Aid Clinical Therapy
Source: Oxid Med Cell Longev. 2022 Feb 27;2022:9039732. doi: 10.1155/2022/9039732 (PMC8898770; doi:10.1155/2022/9039732)
Supplement: Supplementary Materials — Figure S1-S4 with corresponding legends (.docx file) and Table S1-S3 (.pdf files) were uploaded in the Supplemental Files. [file 9039732.f1.zip › Table S2 (1).pdf]

gene	r	p	direct
SLPI	0.66930715	3.90E-70	A
SLC4A3	0.66657572	2.23E-69	A
TRNP1	0.66257654	2.77E-68	A
GFPT2	0.65572687	1.90E-66	A
GPRC5A	0.6527254	1.17E-65	A
UCHL1	0.61918084	2.05E-57	A
TMEM158	0.60619809	1.74E-54	A
MOCOS	0.60146039	1.89E-53	A
SEMA3C	0.59428848	6.49E-52	A
IGF2BP2	0.59066274	3.75E-51	A
MAGED4B	0.58498945	5.58E-50	A
BDKRB1	0.58003806	5.64E-49	A
CPXM1	0.57972897	6.50E-49	A
LEF1	0.57172348	2.51E-47	A
IGDCC4	0.57110836	3.31E-47	A
EFNA5	0.56993907	5.60E-47	A
MFI2	0.56950317	6.80E-47	A
RARRES1	0.56632296	2.79E-46	A
HOXB9	0.56349625	9.69E-46	A
SERPINA3	0.56080041	3.14E-45	A
ITPKA	0.56063216	3.37E-45	A
ADAM12	0.56046174	3.63E-45	A
PPP1R1A	0.5600687	4.30E-45	A
CLMP	0.55975278	4.93E-45	A
ROR2	0.55872454	7.69E-45	A
PTGES	0.55207983	1.30E-43	A
PKP3	0.5502997	2.75E-43	A
KCNS1	0.54695095	1.11E-42	A
RAB3B	0.5454443	2.06E-42	A
CDCP1	0.54446473	3.09E-42	A
10-Mar	0.54373872	4.16E-42	A
SLC12A8	0.54249878	6.90E-42	A
SPTBN2	0.53998363	1.92E-41	A
C10orf90	0.53973379	2.12E-41	A
MAT1A	0.53804509	4.19E-41	A
PROM2	0.53585596	1.01E-40	A
SLC38A5	0.53493922	1.45E-40	A
LAMB3	0.53430494	1.86E-40	A
STEAP3	0.53409045	2.03E-40	A
SAA2	0.53212258	4.41E-40	A
ARL14	0.53011855	9.69E-40	A
MOXD1	0.52507196	6.86E-39	A
LOXL1	0.52448442	8.60E-39	A
CILP2	0.52383728	1.10E-38	A
SAA1	0.52174458	2.45E-38	A

KLK13	0.51874782	7.62E-38	A
SIX4	0.51630891	1.90E-37	A
ACTBL2	0.51444045	3.81E-37	A
PI3	0.51271273	7.23E-37	A
FGF7	0.51247334	7.90E-37	A
ERC2	0.51130895	1.21E-36	A
TUBB3	0.51026867	1.78E-36	A
STMN2	0.50852561	3.36E-36	A
CHRDL2	0.50796444	4.12E-36	A
COL11A1	0.50789724	4.22E-36	A
SERPINA5	0.50658698	6.78E-36	A
F3	0.50565319	9.51E-36	A
CDH3	0.50483465	1.28E-35	A
CPEB1	0.50399414	1.73E-35	A
LUM	0.50208275	3.42E-35	A
FAM101A	0.50126243	4.58E-35	A
KRT15	0.49881595	1.09E-34	A
KCNK15	0.49529299	3.74E-34	A
FNDC4	0.4934446	7.11E-34	A
TMC5	0.49337132	7.30E-34	A
PLTP	0.48662077	7.37E-33	A
C14orf132	0.48651795	7.63E-33	A
REEP2	0.48500621	1.27E-32	A
KRT19	0.47747271	1.56E-31	A
GREM1	0.47038112	1.56E-30	A
DNER	0.46949701	2.07E-30	A
COL10A1	0.46935148	2.17E-30	A
PPP2R2C	0.46906372	2.38E-30	A
WISP2	0.4667144	5.02E-30	A
CAPN8	0.46288624	1.68E-29	A
MAGEL2	0.46039831	3.65E-29	A
TCN1	0.45891058	5.78E-29	A
VSTM2L	0.45734359	9.38E-29	A
GYG2	0.45558464	1.61E-28	A
SH3GL3	0.45359163	2.95E-28	A
TGFBI	0.44768982	1.74E-27	A
CCL11	0.44765998	1.76E-27	A
WNT2	0.44762196	1.78E-27	A
FRMD5	0.44679701	2.27E-27	A
DCN	0.44487948	4.01E-27	A
NTNG1	0.44298248	7.00E-27	A
SLC7A5	0.43912195	2.16E-26	A
TMEM61	0.43759189	3.35E-26	A
FLNC	0.4367141	4.32E-26	A
SERPINA4	0.43529184	6.49E-26	A
LINC00460	0.43504691	6.96E-26	A

EPB41L4B	0.43481492	7.43E-26	A
GPR87	0.43414957	8.98E-26	A
TFCP2L1	0.43110047	2.13E-25	A
DUSP9	0.43022145	2.73E-25	A
CYP11A1	0.42449477	1.34E-24	A
KLC3	0.42296573	2.05E-24	A
WFDC10B	0.41902979	5.98E-24	A
EHF	0.41442132	2.06E-23	A
ARSI	0.41127154	4.75E-23	A
PCP4	0.40928354	8.02E-23	A
ZPLD1	0.40344693	3.64E-22	A
FGF9	0.40068179	7.39E-22	A
CST5	0.39478973	3.26E-21	A
CHRDL1	0.39264815	5.55E-21	A
AGR2	0.39192101	6.65E-21	A
RHCG	0.39124463	7.85E-21	A
SCG5	0.37566082	3.30E-19	A
GSG1	0.37028606	1.15E-18	A
PPP1R1B	0.3640816	4.68E-18	A
HOXB13	0.35996185	1.17E-17	A
IGFN1	0.35543118	3.16E-17	A
EPN3	0.35237207	6.13E-17	A
LHX1	0.35188676	6.80E-17	A
LCN2	0.3475819	1.70E-16	A
GRHL2	0.33301047	3.43E-15	A
DMRT2	0.32771191	9.83E-15	A
PRSS22	0.31109347	2.34E-13	A
KLK6	0.2897967	1.03E-11	A
SLC34A1	-0.4004409	7.86E-22	B
ASPG	-0.413817	2.42E-23	B
SLC2A9	-0.4459099	2.95E-27	B
PAQR5	-0.4533473	3.18E-28	B
FRMD1	-0.4769112	1.87E-31	B
UGT2B7	-0.4948411	4.38E-34	B
WDR72	-0.5082992	3.65E-36	B
USH1C	-0.5163146	1.90E-37	B
SLC28A1	-0.5372258	5.81E-41	B
DMGDH	-0.5446387	2.88E-42	B
PANK1	-0.5578637	1.11E-44	B
KL	-0.5610949	2.76E-45	B
ECHDC3	-0.5630384	1.18E-45	B
LRRK2	-0.563577	9.35E-46	B
FREM2	-0.5649524	5.11E-46	B
SPATA18	-0.5708931	3.65E-47	B
ABHD6	-0.5713146	3.02E-47	B
DMRTA1	-0.5767934	2.51E-48	B

C1orf115	-0.5771198	2.16E-48	B
SHMT1	-0.5783943	1.20E-48	B
CRYL1	-0.5786692	1.06E-48	B
KCNJ15	-0.582718	1.62E-49	B
PTH2R	-0.5851426	5.19E-50	B
PBLD	-0.5890351	8.17E-51	B
PXMP2	-0.5941206	7.04E-52	B
LYG1	-0.5983748	8.75E-53	B
SLC3A1	-0.5985071	8.19E-53	B
C11orf54	-0.601498	1.86E-53	B
SLC7A9	-0.6017439	1.64E-53	B
TMEM38B	-0.6035277	6.71E-54	B
ACSM2B	-0.6045789	3.95E-54	B
AVPR1B	-0.6108511	1.61E-55	B
TRIM10	-0.6124421	7.06E-56	B
FMO1	-0.6126376	6.38E-56	B
EHHADH	-0.6156224	1.34E-56	B
LRP2	-0.6160283	1.08E-56	B
C1orf210	-0.6189408	2.33E-57	B
PKHD1	-0.6205082	1.01E-57	B
SLC6A19	-0.6221839	4.11E-58	B
FUT6	-0.6232892	2.27E-58	B
ACSM2A	-0.6259663	5.30E-59	B
SLC22A2	-0.6260534	5.06E-59	B
MAP7	-0.6296361	7.08E-60	B
CLCN5	-0.6301653	5.28E-60	B
ACADL	-0.6305005	4.39E-60	B
LGALS2	-0.6313882	2.68E-60	B
SLC10A2	-0.6363867	1.62E-61	B
C9orf66	-0.638044	6.33E-62	B
DDAH1	-0.6390145	3.64E-62	B
TAL2	-0.6423905	5.21E-63	B
PDZK1	-0.6427757	4.17E-63	B
SLC6A13	-0.6443378	1.68E-63	B
BBOX1	-0.64911	1.01E-64	B
SLC17A3	-0.649112	1.01E-64	B
SLC17A1	-0.6499819	6.04E-65	B
GATM	-0.6505441	4.32E-65	B
DDC	-0.6520024	1.80E-65	B
GLYATL1	-0.653053	9.58E-66	B
SLC13A1	-0.6548164	3.30E-66	B
PHYHIPL	-0.6551392	2.71E-66	B
SMTNL2	-0.6567091	1.04E-66	B
MSRA	-0.6638261	1.27E-68	B
SLC16A9	-0.6680981	8.47E-70	B
AGMAT	-0.6684148	6.91E-70	B

ACE2	-0.6692654	4.01E-70	B
CYP4A22	-0.6715482	9.21E-71	B
SLC22A11	-0.6736525	2.35E-71	B
ACAA2	-0.6744333	1.41E-71	B
NAT8	-0.6752837	8.06E-72	B
SLC25A48	-0.6755743	6.66E-72	B
SCGN	-0.6770561	2.51E-72	B
GBA3	-0.6802087	3.07E-73	B
NPR3	-0.6830994	4.38E-74	B
SLC22A6	-0.6832625	3.92E-74	B
SLC47A1	-0.6879475	1.59E-75	B
TMEM174	-0.6923443	7.40E-77	B
SLC5A10	-0.6969226	2.87E-78	B
HAO2	-0.697599	1.76E-78	B
GIPC2	-0.6978731	1.45E-78	B
TRPM3	-0.7003179	2.47E-79	B
SLC22A12	-0.7027565	4.16E-80	B
MIOX	-0.706001	3.77E-81	B
CYP4A11	-0.7075707	1.17E-81	B
TMEM27	-0.7117751	4.86E-83	B
SLC27A2	-0.7143898	6.53E-84	B
NAT8B	-0.7212749	2.97E-86	B
SLC22A24	-0.7231611	6.58E-87	B
