# Supplementary material for: Comprehensive Characterization of Metabolism-Associated Subtypes of Renal Cell Carcinoma to Aid Clinical Therapy
Source: Oxid Med Cell Longev. 2022 Feb 27;2022:9039732. doi: 10.1155/2022/9039732 (PMC8898770; doi:10.1155/2022/9039732)
Supplement: Supplementary Materials — Figure S1-S4 with corresponding legends (.docx file) and Table S1-S3 (.pdf files) were uploaded in the Supplemental Files. [file 9039732.f1.zip › Table S3 (1).pdf]

samID	pca1.A	pca1.B	MTB.score	subtype
TCGA-BP-4	-28.140798	37.2328463	65.3736447	A
TCGA-A3-3	-30.355431	49.7148093	80.07024	A
TCGA-A3-3	-35.381792	52.9191254	88.3009171	A
TCGA-CJ-5	-32.325356	53.8337894	86.1591452	A
TCGA-B0-5	-32.685028	51.3483112	84.0333388	A
TCGA-B0-5	-25.611636	58.2160136	83.8276496	A
TCGA-B0-4	-28.152298	49.9166838	78.0689817	A
TCGA-CJ-5	-23.780431	57.5503194	81.3307507	A
TCGA-BP-4	-30.115077	54.6382628	84.7533393	A
TCGA-BP-4	-30.8066	61.3409744	92.1475747	A
TCGA-BP-5	-25.388777	57.2115512	82.600328	A
TCGA-BP-4	-30.821396	47.275202	78.0965985	A
TCGA-BP-4	-28.470216	65.7222515	94.1924679	A
TCGA-B0-5	-27.333813	62.2268792	89.5606922	A
TCGA-BP-4	-32.132241	56.2168741	88.3491155	A
TCGA-B0-5	-28.434814	52.4679219	80.9027363	A
TCGA-DV-A	-29.488507	53.044791	82.5332983	A
TCGA-BP-4	-31.929514	60.0547038	91.9842175	A
TCGA-CJ-4	-34.849654	52.3929359	87.2425895	A
TCGA-BP-5	-35.094526	57.5024629	92.5969885	A
TCGA-BP-4	-30.295479	41.0204332	71.3159117	A
TCGA-BP-4	-30.435842	42.5207545	72.9565963	A
TCGA-A3-3	-29.207669	64.4047242	93.6123929	A
TCGA-MW-	-35.272525	56.3386569	91.6111814	A
TCGA-BP-4	-31.107063	51.8790693	82.9861319	A
TCGA-BP-4	-27.30081	55.2949235	82.5957338	A
TCGA-BP-4	-28.549199	33.5827274	62.1319263	A
TCGA-A3-3	-30.559289	60.4177029	90.9769922	A
TCGA-A3-3	-27.625745	62.6680371	90.2937821	A
TCGA-AK-3	-34.265495	52.7424532	87.0079482	A
TCGA-AK-3	-28.71828	58.7303122	87.448592	A
TCGA-A3-3	-29.636529	55.8129524	85.4494816	A
TCGA-B2-5	-26.206633	58.077309	84.283942	A
TCGA-GK-A	-30.821694	52.8779532	83.6996468	A
TCGA-A3-3	-34.482685	57.6319717	92.1146565	A
TCGA-CJ-4	-27.92558	56.7957398	84.7213197	A
TCGA-BP-4	-28.88601	62.4097679	91.2957782	A
TCGA-CW-	-27.070184	62.3300291	89.4002132	A
TCGA-BP-5	-22.94049	56.2660365	79.2065266	A
TCGA-BP-4	-31.708996	51.891197	83.6001934	A
TCGA-B0-4	-30.181053	55.621808	85.8028609	A
TCGA-B2-A	-30.975889	49.7455835	80.7214725	A
TCGA-CJ-4	-30.626046	56.8316535	87.4576992	A
TCGA-B8-A	-32.308135	54.7729497	87.0810852	A
TCGA-AK-3	-29.720579	61.6743042	91.3948831	A

TCGA-BP-5	-32.644782	56.3983511	89.0431331	A
TCGA-CJ-48	-31.321758	59.1420935	90.4638511	A
TCGA-A3-3	-31.997124	58.2782334	90.2753574	A
TCGA-AK-3	-25.711253	52.9422281	78.653481	A
TCGA-B0-48	-30.553031	54.0123054	84.5653362	A
TCGA-BP-49	-31.249545	56.6954559	87.9450008	A
TCGA-A3-3	-34.645748	58.105102	92.75085	A
TCGA-BP-5	-34.851635	44.5604269	79.4120617	A
TCGA-CJ-48	-27.697299	59.3615012	87.0588005	A
TCGA-CJ-48	-33.256379	57.3706476	90.6270264	A
TCGA-CZ-54	-30.969531	49.559811	80.5293419	A
TCGA-B0-48	-33.514324	49.9472422	83.4615662	A
TCGA-CZ-48	-31.357522	57.0139671	88.3714891	A
TCGA-BP-4	-27.766185	54.6416201	82.4078055	A
TCGA-BP-49	-29.405617	56.8951807	86.3007972	A
TCGA-A3-3	-28.204736	54.9370705	83.1418067	A
TCGA-BP-4	-26.675771	54.7534065	81.4291771	A
TCGA-BP-49	-26.569846	56.1070937	82.6769399	A
TCGA-BP-49	-32.75757	51.8359047	84.593475	A
TCGA-B8-41	-28.369131	61.1992719	89.5684029	A
TCGA-B0-40	-28.385028	54.9913841	83.3764124	A
TCGA-CZ-48	-29.599549	61.2555556	90.8551049	A
TCGA-B0-48	-32.51442	58.62243	91.1368498	A
TCGA-BP-47	-27.456909	57.840013	85.2969222	A
TCGA-BP-47	-27.986082	57.0707901	85.0568723	A
TCGA-A3-3	-35.695218	52.1561736	87.8513915	A
TCGA-B0-51	-29.945247	58.97774	88.9229871	A
TCGA-BP-4	-34.236137	54.3826039	88.6187405	A
TCGA-A3-A	-30.928129	60.630099	91.5582285	A
TCGA-B0-50	-27.038805	58.5175973	85.5564023	A
TCGA-A3-3	-29.702878	56.7392811	86.4421592	A
TCGA-B0-47	-27.523891	39.4131714	66.9370624	A
TCGA-B0-50	-33.726045	54.1393607	87.8654059	A
TCGA-CZ-54	-32.260386	54.1710843	86.4314705	A
TCGA-BP-50	-32.776438	56.2701234	89.0465615	A
TCGA-BP-5	-33.518768	49.593269	83.1120366	A
TCGA-B0-50	-29.727585	51.1515065	80.8790917	A
TCGA-B0-48	-24.404554	53.7855149	78.1900694	A
TCGA-G6-A	-29.808876	49.4415719	79.2504476	A
TCGA-BP-49	-28.646554	53.8273146	82.4738682	A
TCGA-B0-51	-23.88237	40.2793807	64.1617505	A
TCGA-BP-4	-30.833588	48.4904442	79.3240325	A
TCGA-CZ-48	-26.919416	58.5284303	85.4478465	A
TCGA-G6-A	-28.374531	41.9506904	70.3252216	A
TCGA-BP-4	-33.13135	53.5530871	86.6844373	A
TCGA-BP-4	-28.768055	55.9591998	84.7272548	A

TCGA-A3-A	-30.024301	58.0585556	88.0828561	A
TCGA-B0-5	-37.162965	45.2140912	82.3770564	A
TCGA-BP-4	-31.322392	55.8382209	87.1606132	A
TCGA-B8-A	-28.990044	60.9503055	89.9403491	A
TCGA-BP-4	-34.975701	53.9166622	88.8923632	A
TCGA-BP-5	-32.890657	57.8911866	90.7818432	A
TCGA-B0-5	-29.429234	60.7744682	90.2037026	A
TCGA-CJ-4	-33.349338	53.151152	86.50049	A
TCGA-EU-5	-27.975991	63.4427084	91.4186996	A
TCGA-B0-5	-29.859448	52.8612755	82.7207233	A
TCGA-CW-	-26.232049	59.1929862	85.4250353	A
TCGA-A3-3	-29.545222	51.7433599	81.2885817	A
TCGA-CZ-5	-36.081984	60.4736109	96.5555945	A
TCGA-B0-5	-30.859008	46.4483593	77.3073671	A
TCGA-A3-3	-28.31781	59.331496	87.649306	A
TCGA-B8-5	-34.92924	60.3324227	95.2616629	A
TCGA-B4-5	-31.156303	56.2668403	87.4231432	A
TCGA-B8-A	-32.183767	52.4696017	84.653369	A
TCGA-B0-5	-33.035732	53.539715	86.5754469	A
TCGA-B8-5	-34.532025	59.9176962	94.449721	A
TCGA-CJ-4	-28.182567	56.5456482	84.7282154	A
TCGA-B4-5	-33.94735	58.2688427	92.2161926	A
TCGA-CJ-4	-33.281456	58.8688656	92.1503219	A
TCGA-A3-A	-34.131538	59.4754614	93.606999	A
TCGA-BP-4	-32.433052	51.346639	83.7796911	A
TCGA-B0-5	-32.50403	51.0231132	83.5271435	A
TCGA-CJ-4	-29.093675	54.7477273	83.8414021	A
TCGA-B8-5	-33.725225	56.2991632	90.0243881	A
TCGA-CZ-4	-31.782806	64.9675114	96.7503176	A
TCGA-EU-5	-36.553482	55.8038752	92.357357	A
TCGA-B0-4	-35.19887	43.2792255	78.4780959	A
TCGA-CJ-4	-26.781177	58.9679931	85.7491699	A
TCGA-B0-5	-27.748426	49.4855125	77.2339383	A
TCGA-B0-5	-32.819068	58.0602788	90.8793466	A
TCGA-B8-4	-32.632302	63.0048121	95.6371139	A
TCGA-B8-A	-30.612618	55.6779772	86.2905954	A
TCGA-CW-	-32.281235	55.037131	87.3183658	A
TCGA-CJ-4	-24.118589	49.9750582	74.0936468	A
TCGA-B8-4	-27.908031	59.283829	87.1918602	A
TCGA-BP-5	-32.050993	59.2063252	91.2573186	A
TCGA-A3-3	-26.979726	61.8995362	88.879262	A
TCGA-CZ-5	-29.722245	53.1747067	82.8969512	A
TCGA-CJ-4	-33.914672	53.6540588	87.5687313	A
TCGA-B0-4	-28.673294	51.1104969	79.7837911	A
TCGA-CJ-4	-32.324198	52.654473	84.9786708	A
TCGA-B8-5	-33.930038	58.5529144	92.4829529	A

TCGA-CZ-4	-31.737334	57.0518148	88.7891484	A
TCGA-B0-5	-31.504322	53.3725888	84.8769109	A
TCGA-BP-4	-31.284647	58.2940639	89.5787113	A
TCGA-BP-4	-29.388491	61.9784727	91.3669636	A
TCGA-BP-5	-33.177017	49.4725246	82.649542	A
TCGA-B4-5	-35.922192	59.7669482	95.6891406	A
TCGA-B0-5	-27.339879	66.5750752	93.914954	A
TCGA-CZ-5	-30.646469	50.0759401	80.722409	A
TCGA-CZ-5	-31.708667	53.0063705	84.7150371	A
TCGA-B0-5	-22.852193	58.8094437	81.6616366	A
TCGA-DV-5	-28.178909	40.87601	69.0549186	A
TCGA-AK-3	-34.03709	51.9238257	85.9609153	A
TCGA-CZ-5	-36.104903	42.2091561	78.3140588	A
TCGA-CZ-5	-33.103904	49.5729216	82.6768253	A
TCGA-B2-4	-31.127159	54.3698816	85.4970402	A
TCGA-B0-4	-27.613884	60.3486763	87.9625603	A
TCGA-T7-A	-19.65919	47.8109342	67.4701242	A
TCGA-B0-5	-32.891523	62.5986148	95.4901376	A
TCGA-B0-4	-25.975177	52.7831571	78.7583343	A
TCGA-B2-4	-28.568347	60.5021655	89.0705121	A
TCGA-AK-3	-29.114064	51.3395437	80.4536078	A
TCGA-BP-4	-30.818337	54.4108672	85.2292046	A
TCGA-BP-4	-23.464067	56.5621184	80.0261855	A
TCGA-B0-4	-30.855635	60.9295245	91.7851597	A
TCGA-B0-5	-33.509928	53.639797	87.1497249	A
TCGA-BP-4	-25.208313	48.6130796	73.8213926	A
TCGA-BP-5	-31.293895	54.8404018	86.1342964	A
TCGA-A3-A	-29.975307	53.931394	83.9067007	A
TCGA-BP-4	-24.827083	49.5014403	74.3285234	A
TCGA-B0-5	-34.160662	56.4580011	90.6186631	A
TCGA-BP-5	-32.280603	55.5281165	87.80872	A
TCGA-BP-4	-32.235116	57.0351732	89.2702889	A
TCGA-B2-5	-31.760277	44.8995861	76.6598627	A
TCGA-BP-4	-33.542539	56.9304672	90.4730063	A
TCGA-BP-5	-29.409906	37.1962078	66.6061136	A
TCGA-B0-4	-25.76325	52.6517823	78.4150319	A
TCGA-A3-3	-28.774738	52.5478077	81.3225453	A
TCGA-BP-5	-29.703965	58.1207877	87.8247523	A
TCGA-A3-3	-35.618024	59.4289731	95.0469973	A
TCGA-CJ-4	-35.339983	54.091938	89.4319212	A
TCGA-CZ-5	-30.220019	56.7628691	86.9828886	A
TCGA-B4-5	-34.657303	53.5214551	88.1787577	A
TCGA-B0-5	-32.540673	57.6835964	90.2242694	A
TCGA-BP-5	-34.108867	61.2182428	95.3271097	A
TCGA-B0-5	-33.520383	52.6278567	86.1482401	A
TCGA-A3-A	-31.958367	57.2940636	89.2524307	A

TCGA-CZ-5	-33.299334	52.8308413	86.1301757	A
TCGA-BP-5	-37.709007	56.4694091	94.1784159	A
TCGA-CZ-5	-30.379157	55.3161581	85.6953152	A
TCGA-B0-5	-34.574558	43.3016695	77.876227	A
TCGA-BP-4	-25.117832	47.1630153	72.2808476	A
TCGA-CZ-5	-33.700559	57.5100917	91.2106502	A
TCGA-B0-5	-33.481103	58.3678547	91.848958	A
TCGA-B2-3	-26.95494	45.266998	72.2219376	A
TCGA-CZ-5	-39.994285	46.9547138	86.9489988	A
TCGA-A3-3	-27.83846	60.021055	87.8595148	A
TCGA-EU-5	-32.304583	59.7379444	92.0425278	A
TCGA-BP-5	-30.911544	51.776831	82.6883754	A
TCGA-BP-5	-33.578598	50.0336585	83.6122565	A
TCGA-CJ-4	-33.726715	62.7250088	96.4517236	A
TCGA-A3-3	-34.986893	41.1291622	76.1160552	A
TCGA-BP-5	-35.014103	60.0230262	95.0371295	A
TCGA-CW-	-33.639829	64.4246478	98.0644764	A
TCGA-A3-3	-31.574235	52.4797816	84.0540168	A
TCGA-A3-3	-29.444855	55.803612	85.2484672	A
TCGA-CJ-4	-32.815894	58.6813896	91.4972831	A
TCGA-B8-A	-26.901064	48.4862325	75.3872961	A
TCGA-G6-A	-25.14653	54.0022082	79.1487387	A
TCGA-CZ-5	-33.601183	61.1260592	94.7272421	A
TCGA-BP-4	-27.669442	48.8053935	76.4748351	A
TCGA-BP-4	-30.477833	52.8162862	83.2941188	A
TCGA-BP-5	-34.899762	58.3873575	93.2871195	A
TCGA-BP-5	-35.383874	48.8370114	84.2208855	A
TCGA-B0-5	-25.295081	59.1508793	84.4459605	A
TCGA-B4-5	-30.161298	63.4945741	93.6558721	A
TCGA-BP-4	-31.211709	37.3433544	68.5550636	A
TCGA-A3-3	-30.168947	61.6599423	91.8288896	A
TCGA-CZ-5	-33.271234	54.3792551	87.6504887	A
TCGA-CJ-4	-30.547818	52.0028118	82.5506296	A
TCGA-B0-4	-31.439104	54.6523042	86.0914083	A
TCGA-BP-4	-30.977351	53.9002156	84.8775666	A
TCGA-BP-4	-33.90556	51.5836216	85.489182	A
TCGA-BP-5	-29.451038	36.1312801	65.582318	A
TCGA-B8-4	-30.599201	51.9131662	82.5123674	A
TCGA-B0-5	-33.466798	57.2903062	90.757104	A
TCGA-B0-5	-33.873702	48.9820891	82.8557915	A
TCGA-CJ-4	-32.87731	58.9621943	91.8395041	A
TCGA-B8-A	-35.485523	51.8971438	87.3826667	A
TCGA-AK-3	-28.688397	62.4533356	91.1417321	A
TCGA-CJ-5	-27.481617	51.4520961	78.9337127	A
TCGA-BP-4	-29.972155	55.2391533	85.2113078	A
TCGA-CZ-4	-32.845264	60.7616419	93.6069063	A

TCGA-A3-3[illegible]	-33.525058	53.9586704	87.483728	A
TCGA-CW-[illegible]	-26.603445	61.955765	88.5592104	A
TCGA-BP-5[illegible]	-31.146303	62.4874684	93.6337709	A
TCGA-CJ-48	-31.079199	54.1511903	85.230389	A
TCGA-A3-3[illegible]	-33.254179	53.3229896	86.5771681	A
TCGA-B0-5[illegible]	-29.294244	61.1931074	90.4873513	A
TCGA-CZ-5[illegible]	-32.060836	56.3483774	88.4092137	A
TCGA-A3-3[illegible]	-29.989359	56.7924652	86.781824	A
TCGA-A3-3[illegible]	-30.38399	56.7932682	87.1772585	A
TCGA-CJ-48	-32.710179	61.9296853	94.6398639	A
TCGA-B0-4[illegible]	-26.208489	34.0514597	60.2599487	A
TCGA-BP-4[illegible]	-29.320202	42.98953	72.309732	A
TCGA-CJ-48	-31.072882	48.6013356	79.6742173	A
TCGA-CJ-60	-30.260218	64.8765153	95.1367329	A
TCGA-CJ-56	-30.478143	51.9675563	82.4456996	A
TCGA-BP-4[illegible]	-32.111743	57.0565404	89.1682834	A
TCGA-BP-4[illegible]	-30.834517	59.0397479	89.8742653	A
TCGA-A3-A	-33.803325	60.070206	93.8735308	A
TCGA-CZ-5[illegible]	-38.207141	58.3143669	96.5215081	A
TCGA-B8-4[illegible]	-34.203836	59.4203325	93.6241689	A
TCGA-CZ-5[illegible]	-29.782983	50.9467531	80.7297366	B
TCGA-CJ-60	-27.08642	41.1950719	68.2814917	B
TCGA-CJ-48	-28.18701	51.3600594	79.5470698	B
TCGA-CW-[illegible]	-25.978633	49.4396682	75.4183017	B
TCGA-BP-4[illegible]	-24.780442	48.0842319	72.8646734	B
TCGA-BP-4[illegible]	-21.230537	53.2778163	74.5083531	B
TCGA-CJ-56	-25.476994	45.3931299	70.8701243	B
TCGA-AK-3	-25.769731	50.938838	76.7085693	B
TCGA-B0-5[illegible]	-28.092675	45.5489624	73.6416376	B
TCGA-BP-4[illegible]	-23.162103	47.533751	70.6958536	B
TCGA-BP-4[illegible]	-29.292552	54.4029259	83.6954777	B
TCGA-BP-4[illegible]	-25.028128	49.9961926	75.0243211	B
TCGA-BP-5[illegible]	-23.476225	49.3733747	72.8495999	B
TCGA-B2-5[illegible]	-25.386931	41.4317142	66.8186456	B
TCGA-EU-5[illegible]	-26.531077	40.2085086	66.7395858	B
TCGA-CJ-60	-25.432025	49.2726935	74.7047185	B
TCGA-B8-5[illegible]	-25.832205	50.2702229	76.1024277	B
TCGA-CJ-56	-22.023929	58.7071786	80.7311076	B
TCGA-CW-[illegible]	-19.653088	58.0613025	77.7143906	B
TCGA-BP-5[illegible]	-26.763804	46.2165096	72.9803134	B
TCGA-B2-4[illegible]	-27.910026	52.8426273	80.7526529	B
TCGA-CJ-48	-27.312901	49.2748008	76.5877013	B
TCGA-B0-4[illegible]	-23.939851	49.0998465	73.039697	B
TCGA-CJ-56	-22.362439	47.2987678	69.6612067	B
TCGA-A3-3[illegible]	-21.644431	52.2776079	73.9220384	B
TCGA-CZ-5[illegible]	-25.875019	57.5993889	83.4744077	B

TCGA-CJ-56	-27.903918	58.4314157	86.3353339	B
TCGA-BP-5	-33.914262	50.2495159	84.163778	B
TCGA-BP-4	-30.642904	57.2547911	87.8976951	B
TCGA-B0-48	-19.889733	45.8874339	65.7771671	B
TCGA-B8-5	-28.033071	50.9065707	78.9396417	B
TCGA-CJ-46	-27.247543	46.1781681	73.4257111	B
TCGA-CW-5	-24.720843	60.7074033	85.4282467	B
TCGA-BP-4	-26.862425	50.2743276	77.1367528	B
TCGA-CZ-5	-25.660666	45.9101779	71.5708441	B
TCGA-BP-4	-11.15135	44.3393839	55.490734	B
TCGA-BP-4	-25.578473	50.9139988	76.4924719	B
TCGA-BP-4	-24.977529	47.4603361	72.4378649	B
TCGA-A3-3	-25.857059	46.9054804	72.7625396	B
TCGA-B0-48	-24.7303	46.2352268	70.9655268	B
TCGA-DV-5	-32.403802	43.6991344	76.1029368	B
TCGA-B0-5	-16.229563	47.1950257	63.4245884	B
TCGA-B0-5	-27.601134	46.2481611	73.8492949	B
TCGA-DV-5	-28.870837	53.6108977	82.4817348	B
TCGA-BP-4	-25.806721	46.0747832	71.8815038	B
TCGA-BP-4	-26.596028	51.7618367	78.357865	B
TCGA-AK-3	-19.666193	55.6680823	75.3342755	B
TCGA-6D-A	-22.149855	49.3403607	71.4902157	B
TCGA-DV-5	-14.658658	41.0732275	55.7318853	B
TCGA-A3-3	-15.946023	47.1179553	63.0639784	B
TCGA-CJ-56	-22.32046	56.7620571	79.0825167	B
TCGA-B0-48	-29.539528	46.0220051	75.5615335	B
TCGA-B0-48	-25.755901	46.5464358	72.3023371	B
TCGA-BP-4	-22.485616	49.7313659	72.2169815	B
TCGA-CJ-49	-23.779409	50.9839432	74.7633519	B
TCGA-BP-4	-22.023945	56.9369371	78.9608824	B
TCGA-A3-3	-24.265886	51.6528609	75.9187473	B
TCGA-AK-3	-26.067049	52.5157602	78.5828094	B
TCGA-A3-A	-21.650759	51.1141567	72.7649161	B
TCGA-BP-4	-25.235949	54.186494	79.4224433	B
TCGA-MM-	-26.364042	48.4390835	74.8031256	B
TCGA-B8-4	-21.392731	52.6934407	74.0861722	B
TCGA-B0-5	-16.283609	60.2336073	76.5172165	B
TCGA-BP-4	-30.586822	50.022529	80.6093507	B
TCGA-AK-3	-22.849718	53.8941832	76.7439014	B
TCGA-BP-4	-26.695808	45.366449	72.0622573	B
TCGA-BP-4	-11.167873	48.8086744	59.9765469	B
TCGA-CJ-49	-29.889115	48.6905151	78.5796302	B
TCGA-CJ-49	-23.077549	50.9035348	73.9810837	B
TCGA-A3-3	-20.117546	50.7696983	70.887244	B
TCGA-CW-5	-25.670813	51.0933204	76.7641338	B
TCGA-AK-3	-24.358789	51.5134288	75.872218	B

TCGA-3Z-A	-24.318077	53.5132002	77.8312774	B
TCGA-CJ-48	-24.802457	43.664417	68.4668735	B
TCGA-BP-4	-30.269812	46.9514575	77.221269	B
TCGA-B0-50	-22.333162	47.8650266	70.198189	B
TCGA-CJ-46	-29.328596	53.3861166	82.7147128	B
TCGA-AK-3	-24.07103	53.3512189	77.4222485	B
TCGA-BP-5	-29.337915	49.3754841	78.7133993	B
TCGA-BP-4	-23.183543	52.5456753	75.7292183	B
TCGA-B0-4	-26.954413	51.8249743	78.7793872	B
TCGA-BP-4	-24.746731	46.2971547	71.0438856	B
TCGA-B0-48	-25.207761	43.7204962	68.9282573	B
TCGA-BP-4	-24.506871	48.8183228	73.3251935	B
TCGA-BP-4	-28.057898	51.0004897	79.0583877	B
TCGA-DV-5	-19.028506	59.7173519	78.7458574	B
TCGA-A3-3	-18.767755	62.2825829	81.0503374	B
TCGA-BP-4	-32.300572	54.2977923	86.5983641	B
TCGA-B0-4	-23.042108	44.8845844	67.9266922	B
TCGA-BP-4	-24.265445	44.6779611	68.9434059	B
TCGA-B2-56	-30.812515	54.0594002	84.8719153	B
TCGA-B0-58	-25.610195	54.1766713	79.7868663	B
TCGA-B4-58	-18.012102	48.6205897	66.6326917	B
TCGA-CJ-46	-32.334054	43.9539277	76.287982	B
TCGA-DV-5	-16.362235	53.7935854	70.1558201	B
TCGA-CZ-4	-26.867399	53.7351128	80.6025119	B
TCGA-B0-5	-26.493519	51.195605	77.6891241	B
TCGA-BP-4	-20.341874	46.2526371	66.594511	B
TCGA-B0-48	-26.962359	49.346051	76.3084096	B
TCGA-BP-4	-23.959263	60.3800894	84.3393521	B
TCGA-CZ-4	-23.91354	44.0858893	67.9994292	B
TCGA-B0-48	-25.98766	54.1084186	80.0960786	B
TCGA-CZ-5	-27.055103	54.6636664	81.7187698	B
TCGA-CZ-4	-26.690701	52.7082501	79.3989506	B
TCGA-B4-58	-24.610747	57.0295711	81.640318	B
TCGA-AK-3	-18.198761	47.418771	65.6175324	B
TCGA-CW-5	-25.938692	42.4335417	68.3722332	B
TCGA-BP-5	-24.231583	54.0847796	78.3163623	B
TCGA-CW-5	-13.161957	56.3303169	69.4922743	B
TCGA-A3-3	-25.007781	50.2288791	75.2366602	B
TCGA-CZ-5	-20.544485	50.2692302	70.8137153	B
TCGA-CJ-49	-28.895829	43.6195975	72.5154264	B
TCGA-B0-46	-26.182225	48.0349543	74.2171796	B
TCGA-B8-A	-24.535774	42.4737798	67.0095541	B
TCGA-B8-5	-15.804112	53.4626344	69.2667463	B
TCGA-CZ-5	-29.040364	50.4454615	79.4858259	B
TCGA-CW-5	-21.880985	61.513902	83.394887	B
TCGA-CJ-56	-18.888192	57.3093519	76.1975443	B

TCGA-CJ-56	-19.581715	39.0680904	58.6498049	B
TCGA-B8-46	-28.220877	54.6650741	82.8859509	B
TCGA-DV-A	-25.857866	46.9599073	72.8177737	B
TCGA-B8-55	-26.152069	52.0946245	78.2466935	B
TCGA-A3-3:	-31.563136	46.9663768	78.5295126	B
TCGA-CJ-46	-21.388648	56.3931357	77.7817838	B
TCGA-B8-5:	-32.715385	49.7196572	82.4350421	B
TCGA-B0-48	-27.2235	42.8686023	70.0921027	B
TCGA-B8-5:	-17.719449	56.7922709	74.51172	B
TCGA-A3-A	-23.504839	53.7820062	77.2868449	B
TCGA-B0-47	-25.760563	50.4529331	76.2134958	B
TCGA-B0-49	-21.822315	52.4700944	74.2924092	B
TCGA-AS-37	-27.892066	57.9630142	85.8550798	B
TCGA-BP-49	-31.848756	45.3718736	77.2206301	B
TCGA-DV-5	-25.083331	53.049059	78.1323899	B
TCGA-A3-3:	-22.048287	51.4907516	73.5390385	B
TCGA-B0-57	-22.805229	55.3052166	78.110446	B
TCGA-A3-3:	-28.612067	46.6550412	75.267108	B
TCGA-DV-5	-24.081595	40.5681237	64.6497184	B
TCGA-B0-56	-18.274687	50.7825775	69.0572643	B
TCGA-BP-5	-26.14124	53.1383949	79.2796352	B
TCGA-CJ-48	-24.975739	48.7973447	73.7730838	B
TCGA-BP-4	-23.592944	44.4543814	68.047325	B
TCGA-BP-49	-27.829326	49.9108981	77.7402237	B
TCGA-CJ-48	-29.520239	50.5578684	80.0781072	B
TCGA-CZ-59	-31.117445	42.0191405	73.1365852	B
TCGA-BP-4:	-24.203762	48.7619298	72.9656913	B
TCGA-CZ-54	-28.584729	36.6841979	65.2689268	C
TCGA-CZ-4:	-25.826892	17.1780107	43.004903	C
TCGA-B0-5:	-17.047441	21.5124048	38.559846	C
TCGA-BP-4:	-20.781125	4.13725997	24.9183849	C
TCGA-B2-40	-21.329497	38.8677373	60.1972339	C
TCGA-MM-,	-22.861097	44.5851371	67.4462341	C
TCGA-B0-50	-27.58712	37.4093352	64.9964551	C
TCGA-BP-4	-31.389992	33.3906205	64.7806121	C
TCGA-BP-5	-32.437046	37.1425414	69.5795872	C
TCGA-B0-46	-25.189458	3.2982936	28.487752	C
TCGA-CJ-49	-25.244791	32.4748459	57.7196374	C
TCGA-B2-39	-13.39391	4.64901654	18.0429265	C
TCGA-CJ-49	-21.303234	40.3160308	61.6192644	C
TCGA-AK-3	-18.689038	2.37776476	21.066803	C
TCGA-MM-,	-28.757642	28.4507808	57.2084226	C
TCGA-B0-5:	-19.935511	13.8161114	33.7516221	C
TCGA-B0-54	-27.990266	36.6261822	64.6164482	C
TCGA-CJ-48	-22.3794	36.5159455	58.8953455	C
TCGA-AK-3	-15.834274	3.19442669	19.0287008	C

TCGA-CZ-5	-23.524672	29.9111459	53.4358176	C
TCGA-B0-4	-19.271295	-6.6765051	12.5947897	C
TCGA-AK-3	-14.71126	1.55661979	16.2678797	C
TCGA-BP-4	-21.828957	15.7148163	37.5437733	C
TCGA-B4-5	-20.544596	24.5441669	45.0887629	C
TCGA-B0-4	-24.444512	40.392055	64.8365673	C
TCGA-A3-3	-32.046279	35.7314224	67.7777012	C
TCGA-B0-5	-26.399283	34.262055	60.661338	C
TCGA-CJ-4	-23.010059	37.259605	60.2696641	C
TCGA-B8-A	-19.840107	20.1517908	39.9918976	C
TCGA-CJ-4	-25.236915	31.5014187	56.7383338	C
TCGA-BP-4	-28.674873	26.4372636	55.1121369	C
TCGA-CJ-4	-18.251887	32.3009983	50.5528856	C
TCGA-AK-3	-28.786163	36.0043301	64.7904935	C
TCGA-BP-5	-31.669833	39.2326299	70.9024629	C
TCGA-BP-4	-23.437589	34.9163161	58.3539054	C
TCGA-A3-3	-29.795705	19.8392995	49.6350043	C
TCGA-B0-4	-25.121507	18.3122524	43.4337598	C
TCGA-B0-5	-23.3768	24.9961714	48.3729712	C
TCGA-B0-4	-25.629549	31.2586122	56.8881611	C
TCGA-CJ-6	-28.381898	41.4678187	69.8497164	C
TCGA-DV-5	-21.106168	22.1788069	43.2849753	C
TCGA-B0-5	-23.413036	29.053648	52.4666841	C
TCGA-BP-4	-25.491646	39.9505509	65.4421972	C
TCGA-B8-4	-29.85645	21.9878168	51.8442664	C
TCGA-CJ-5	-29.78911	33.028654	62.8177638	C
TCGA-BP-4	-27.206152	30.3014303	57.5075824	C
TCGA-CJ-4	-27.556023	34.5378816	62.0939043	C
TCGA-B0-4	-25.73565	25.9541196	51.6897695	C
TCGA-AK-3	-15.520938	5.7326267	21.253565	C
TCGA-BP-4	-28.335729	37.2721087	65.607838	C
TCGA-CW-6	-32.537338	26.485815	59.0231527	C
TCGA-B0-4	-31.398128	29.5746455	60.9727734	C
TCGA-BP-4	-12.559465	1.91262847	14.4720937	C
TCGA-BP-5	-34.404765	21.8339577	56.2387227	C
TCGA-B0-4	-24.7585	24.0817486	48.8402481	C
TCGA-AK-3	-15.2454	1.77973781	17.0251373	C
TCGA-B0-4	-30.781359	39.0155503	69.7969089	C
TCGA-BP-4	-30.831909	34.3576692	65.1895783	C
TCGA-B8-5	-23.182567	24.6903469	47.8729141	C
TCGA-AK-3	-22.248064	19.2347364	41.4828003	C
TCGA-BP-4	-18.526601	19.3215691	37.8481699	C
TCGA-B0-5	-30.742421	41.8746739	72.6170948	C
TCGA-BP-5	-24.773344	35.5036556	60.2769998	C
TCGA-B0-5	-15.092784	18.8345816	33.9273655	C
TCGA-CJ-4	-32.925243	33.1587092	66.0839526	C

TCGA-B0-4(	-13.478583	7.94347398	21.4220574	C
TCGA-B0-4(	-24.920768	-3.6644396	21.256328	C
TCGA-CZ-4!	-28.593298	34.1145697	62.7078677	C
TCGA-DV-A	-18.473016	7.71691934	26.1899357	C
TCGA-AK-3	-33.752132	38.4407438	72.1928762	C
TCGA-BP-4!	-30.674156	34.9412721	65.6154286	C
TCGA-BP-4	-22.590477	6.52087677	29.111354	C
TCGA-B0-4	-29.723953	26.0941303	55.8180831	C
TCGA-CJ-48	-26.202347	38.3753003	64.577647	C
TCGA-B0-4!	-24.380762	28.2572667	52.6380291	C
TCGA-B0-4(	-27.306351	-1.8055307	25.5008203	C
TCGA-B0-5(	-24.768421	14.5902065	39.3586274	C
TCGA-AK-3	-12.103638	30.2684953	42.3721333	C
TCGA-B0-4	-26.303294	34.6740561	60.9773503	C
TCGA-CJ-60	-32.695355	39.7751006	72.4704554	C
TCGA-CZ-5	-35.060222	24.8627583	59.9229802	C
TCGA-B8-5	-33.233943	25.1486137	58.3825567	C
TCGA-B0-4!	-26.362297	36.5337034	62.8960009	C
TCGA-CJ-48	-24.362935	25.0313654	49.3943005	C
TCGA-CW-(	-22.837372	40.2269514	63.0643234	C
TCGA-CJ-48	-29.316282	47.5102825	76.8265648	C
TCGA-CJ-46	-25.263269	41.8640755	67.1273441	C
TCGA-BP-4	-25.920344	36.2167349	62.137079	C
TCGA-BP-5	-28.06837	35.9336083	64.0019787	C
TCGA-CJ-56	-33.545632	42.1673046	75.7129366	C
TCGA-BP-4	-30.867868	34.7013331	65.569201	C
TCGA-A3-3	-19.853034	34.8270941	54.6801282	C
TCGA-B4-5!	-33.019812	42.0426055	75.0624174	C
TCGA-AS-3	-13.78074	24.7024734	38.4832137	C
TCGA-A3-3	-34.026834	40.3726713	74.3995052	C
TCGA-BP-5	-28.648975	36.2287715	64.8777461	C
TCGA-B8-A	-31.590955	41.6001183	73.1910736	C
TCGA-CJ-56	-28.353908	41.1184242	69.4723319	C
TCGA-BP-4	-15.930029	15.7196108	31.6496394	C
TCGA-BP-4	-27.540916	27.7296191	55.2705349	C
TCGA-B2-5(	-19.781191	25.7473266	45.5285171	C
TCGA-B0-5	-32.986934	36.9175722	69.904506	C
TCGA-B8-5	-28.375035	33.0671135	61.4421482	C
TCGA-BP-4	-20.143627	19.0511929	39.1948204	C
TCGA-B8-4(	-29.33814	26.9326657	56.270806	C
TCGA-BP-5	-33.922335	35.2072391	69.1295739	C
TCGA-CJ-60	-29.776877	34.5426746	64.319552	C
TCGA-B0-4!	-26.871129	31.5262591	58.3973878	C
TCGA-BP-4	-18.704617	20.4830894	39.1877067	C
TCGA-BP-4!	-18.448721	25.8083668	44.2570876	C
TCGA-AK-3	-26.29322	39.0922524	65.3854724	C

TCGA-B8-A	-29.303617	34.7274697	64.0310871	C
TCGA-B0-5:	-13.172876	3.27458154	16.4474575	C
TCGA-A3-3:	-16.190838	-4.2414671	11.9493713	C
TCGA-B0-5(	-16.158254	17.2862391	33.444493	C
TCGA-B8-4(	-16.443099	6.16331046	22.6064095	C
TCGA-G6-A	-31.379596	23.1704698	54.5500663	C
TCGA-CJ-49	-25.839031	29.5886525	55.4276832	C
TCGA-BP-5	-26.269491	31.4733537	57.7428449	C
TCGA-A3-3:	-13.327133	3.25175261	16.5788853	C
TCGA-BP-4	-25.269859	24.7445921	50.0144513	C
TCGA-B0-48	-22.748256	13.6141352	36.3623915	C
TCGA-BP-4:	-22.503856	45.2836355	67.7874919	C
TCGA-B0-5(	-26.962134	34.4212996	61.3834335	C
TCGA-CZ-4	-27.015784	5.12463481	32.1404185	C
TCGA-CJ-46	-24.151183	32.71042	56.861603	C
TCGA-B0-5	-25.315161	33.3934702	58.7086314	C
TCGA-B0-48	-32.87208	30.9112948	63.7833748	C
TCGA-CZ-4	-23.884986	42.0611319	65.9461175	C
TCGA-B0-5	-31.186191	35.4223427	66.6085336	C
TCGA-BP-4	-20.509972	43.681217	64.1911892	C
TCGA-CJ-49	-25.739483	39.5916878	65.331171	C
TCGA-B0-48	-20.846694	3.03407175	23.8807662	C
TCGA-CW-(	-19.580264	16.5333852	36.1136493	C
TCGA-BP-4	-17.306441	31.6139886	48.9204299	C
TCGA-BP-4	-35.301933	17.0148782	52.3168112	C
